# Proportion and characteristics of secondary progressive multiple sclerosis in five European registries using objective classifiers

**DOI:** 10.1177/20552173231153557

**Published:** 2023-02-16

**Authors:** Lars Forsberg, Tim Spelman, Pernilla Klyve, Ali Manouchehrinia, Ryan Ramanujam, Elena Mouresan, Jiri Drahota, Dana Horakova, Hanna Joensen, Luigi Pontieri, Melinda Magyari, David Ellenberger, Alexander Stahmann, Jeff Rodgers, James Witts, Rod Middleton, Richard Nicholas, Vladimir Bezlyak, Nicholas Adlard, Thomas Hach, Carol Lines, Sandra Vukusic, Merja Soilu-Hänninen, Anneke van der Walt, Helmut Butzkueven, Pietro Iaffaldano, Maria Trojano, Anna Glaser, Jan Hillert, on behalf of the SPMS Research Collaboration Network

**Affiliations:** Department of Clinical Neuroscience, Karolinska Institutet, Stockholm, Sweden; Department of Clinical Neuroscience, Karolinska Institutet, Stockholm, Sweden; Department of Clinical Neuroscience, Center for Molecular Medicine, Karolinska Institutet, Stockholm, Sweden; Department of Clinical Neuroscience, Karolinska Institutet, Stockholm, Sweden; Department of Mathematics, Royal Institute of Technology, Stockholm, Sweden; Department of Clinical Neuroscience, Karolinska Institutet, Stockholm, Sweden; Czech National Multiple Sclerosis ReMuS, IMPULS Endowment Fund, Prague, Czech Republic; First Faculty of Medicine and General University Hospital, Department of Neurology and Center of Clinical Neuroscience, Charles University in Prague, Prague, Czech Republic; First Faculty of Medicine and General University Hospital, Department of Neurology and Center of Clinical Neuroscience, Charles University in Prague, Prague, Czech Republic; The Danish Multiple Sclerosis Registry, Copenhagen University Hospital, Copenhagen, Denmark; The Danish Multiple Sclerosis Registry, Copenhagen University Hospital, Copenhagen, Denmark; Danish Multiple Sclerosis Center, Copenhagen University Hospital, Copenhagen, Denmark; MS Forschungs- und Projektentwicklungs-gGmbH, Hannover, Germany; Swansea University Medical School, Swansea, UK; Swansea University Medical School, Swansea, UK; Department of Cellular and Molecular Neuroscience, Imperial College London, London, UK; Novartis Pharma AG, Basel, Switzerland; Hôpital Neurologique, Service de Neurologie A, the European Database for Multiple Sclerosis (EDMUS), Coordinating Center and INSERM U 433, Lyon, France; Division of Clinical Neurosciences, University Hospital and University of Turku, Turku, Finland; Department of Neuroscience, Central Clinical School, Monash University, Melbourne, Australia; Department of Basic Medical Sciences, Neurosciences and Sense Organs, University of Bari “Aldo Moro”, Bari, Italy; Department of Clinical Neuroscience, Karolinska Institutet, Stockholm, Sweden

**Keywords:** Multiple sclerosis, progression, disease course, SPMS, disease-modifying treatments

## Abstract

**Background:**

To assign a course of secondary progressive multiple sclerosis (MS) (SPMS) may be difficult and the proportion of persons with SPMS varies between reports. An objective method for disease course classification may give a better estimation of the relative proportions of relapsing–remitting MS (RRMS) and SPMS and may identify situations where SPMS is under reported.

**Materials and methods:**

Data were obtained for 61,900 MS patients from MS registries in the Czech Republic, Denmark, Germany, Sweden, and the United Kingdom (UK), including date of birth, sex, SP conversion year, visits with an Expanded Disability Status Scale (EDSS) score, MS onset and diagnosis date, relapses, and disease-modifying treatment (DMT) use. We included RRMS or SPMS patients with at least one visit between January 2017 and December 2019 if ≥ 18 years of age. We applied three objective methods: A set of SPMS clinical trial inclusion criteria (“EXPAND criteria”) modified for a real-world evidence setting, a modified version of the MSBase algorithm, and a decision tree-based algorithm recently published.

**Results:**

The clinically assigned proportion of SPMS varied from 8.7% (Czechia) to 34.3% (UK). Objective classifiers estimated the proportion of SPMS from 15.1% (Germany by the EXPAND criteria) to 58.0% (UK by the decision tree method). Due to different requirements of number of EDSS scores, classifiers varied in the proportion they were able to classify; from 18% (UK by the MSBase algorithm) to 100% (the decision tree algorithm for all registries). Objectively classified SPMS patients were older, converted to SPMS later, had higher EDSS at index date and higher EDSS at conversion. More objectively classified SPMS were on DMTs compared to the clinically assigned.

**Conclusion:**

SPMS appears to be systematically underdiagnosed in MS registries. Reclassified patients were more commonly on DMTs.

## Introduction

In 1868, Jean-Martin Charcot recognized that multiple sclerosis (MS) may follow a progressive course. However, it was not until 1955 that McAlpine recognized two forms of progression; primary progressive MS (PPMS) and secondary progressive MS (SPMS) that follows an initially relapsing–remitting MS (RRMS) course. Initially, 85% of all patients are classified as RRMS but many will eventually transition to a progressive course (SPMS).^1,2^

Subsequently, four clinical subtypes were defined in 1996 based on a survey of MS experts.^3^ Here, SPMS was defined as an “initial relapsing remitting disease course followed by progression with or without occasional relapses, minor remissions, and plateaus”. The fourth subtype, called progressive relapsing MS, was later dropped.^4^

From a pathogenetic and clinical perspective, RRMS is characterized by focal inflammatory demyelinating lesions in white matter, whereas SPMS is characterized by diffuse injury of white matter, cortical demyelination, and grey matter atrophy.^5,6^ However, no biological distinction or clinical marker identifies the transition to SPMS and the assignment of RRMS or SPMS is based on a patients’ history and disability status. Also, cortical lesions and atrophy may also be present in early RRMS, indicating that SPMS transition is a slow and inherently blurred process.^7,8^ Accordingly, the traditional dichotomy between RRMS and SPMS has been challenged in recent years but remains a basis for regulatory decisions, most importantly in labels for disease-modifying treatments (DMTs).^9,10^

In clinical trials, where a well-defined study cohort is key, it is common to include a certain level of disability and recent progression in the inclusion criteria of SPMS. For instance, in the siponimod pivotal study (EXPAND), the criteria were an Expanded Disability Status Scale (EDSS) score of 3.0–6.5, a documented EDSS progression 2 years prior to study enrollment without evidence of relapse in the past 3 months, and a previous clinical assignment of SPMS.^11^ This set of criteria adds objectivity in assigning SPMS but may exclude some true SPMS patients.

Clinical MS cohorts, MS databases, and MS registries have become increasingly important sources of information for epidemiological studies.^12^ However, a lack of consensus on how to practically assign SPMS may question clinically assigned SPMS. In addition, the proportion of SPMS in cohorts of MS patients has varied between reports^13^ and the clinical assignment of SPMS is sometimes uncertain, in particular at its early stage.^10^ This has prompted initiatives to define objective approaches to assign SPMS, in order to ensure comparability between studies. The MSBase algorithm was the first proposed objective method and is based on three EDSS observations.^14^ It has also previously been applied in a study regarding risk factors in SPMS,^15^ in a study to identify RRMS patients at risk for SPMS,^16^ and in a comparison with the performance of the neurologist's definition of SPMS.^17^ However, since this algorithm requires three visits to establish a conversion, many patient files in registries lack enough documentation to allow classification. The need for a simpler yet accurate approach recently resulted in the development of a decision tree-based classifier that only relies on age and one EDSS score.^18^ Both these classifiers were optimized and validated against patients with clinically assigned course, showing a high level of concordance with clinical assignment of SPMS, while also being able to reassign clinical outliers.

The aims of this study were to estimate the proportion and characteristics of SPMS patients among RRMS and SPMS patients in five European registries using the clinically assigned MS course and three objective SPMS classifiers; a modified EXPAND criteria for real-world data without a clinical assigned MS course, a modified MSBase algorithm without requiring a functional system (FS) score, and the decision tree classifier.

## Methods

### Study population

Data on clinical and demographic characteristics for 61,950 patients were obtained from MS registries in the United Kingdom (UK) (3890 females, 1171 males),^19^ Czech Republic (8228 females, 3108 males),^20^ Germany (17,371 females, 6598 males),^21^ Denmark (7190 females, 3150 males),^22^ and Sweden (8024 females, 3220 males).^23^ Information on date of birth, sex, SP conversion year (when applicable), visits with EDSS score (WebEDSS in UK), MS onset date, MS diagnosis date, and DMT use were extracted. Visits without EDSS scores were excluded. Inclusion criteria were to have at least one visit during the index period from January 2017 to December 2019 and to have an age ≥ 18 years at the last visit. The last visit was considered the “index date” for each patient. Only patients with either a clinically assigned RRMS or SPMS were included.

### Objective classification methods

Three different objective methods were applied; the inclusion criteria used in the EXPAND study were modified for a real-world evidence setting, a modified version of the MSBase algorithm, and a decision tree-based classifier.

The EXPAND inclusion criteria stem from the phase 3 study on siponimod in SPMS patients^11^ was used for the enrollment of clinically assigned SPMS patients. These criteria required an age of 18–60 years, an EDSS level of 3.0–6.5 at screening, a history of RRMS, no relapses 3 months prior to screening, and a documented EDSS progression 2 years prior to screening. In this study, the criteria were modified to classify patients as RRMS or SPMS based on a similar set of rules, but without a requirement of having a clinically assigned SPMS, relapses, a certain age range, or upper EDSS score. The modified method required a baseline EDSS observation and a follow-up EDSS observation 12–24 months after the baseline to allow classification of patients as RRMS or SPMS. If follow-up EDSS ≥ 3.0 and EDSS change from baseline was either at least 1.0 (if follow-up EDSS < 6.0) or 0.5 (if follow-up EDSS ≥ 6.0), the patient was classified as SPMS but only if future EDSS observations also maintained the EDSS ≥ 3.0 level.

The original MSBase algorithm was selected as the best definition out of 576 different data-derived onset definitions of SPMS.^14^ The definition with the best performance required a minimum EDSS of 4 and a pyramidal FS score of 2, a documented EDSS change of 1.0 from baseline to follow-up if EDSS ≤ 5.5 or an EDSS change of 0.5 if EDSS ≥ 6.0, no relapses 30 days prior to the follow-up visit, and a confirmation of the progression ≥ 3 months after follow-up. There was no requirement of a specific time gap between the baseline and follow-up visits. Instead, all visits after the first baseline visit were considered potential follow-ups. In this study, the algorithm was slightly changed by removing the requirement of pyramidal FS score, since most registries did not have this information. A requirement of no relapses 30 days prior to the confirmation visit was also included to align with the rule of the follow-up visit.

The decision tree classifier was developed with the aim to classify a patient as either RRMS or SPMS using only one EDSS observation and age at that time, using data from the Swedish registry and machine learning methodology for training and data from British Columbia for validation. No follow-up visit was required. For detailed information about the classifier's rules, see publication by Ramanujam et al.^18^

**Figure 1.**
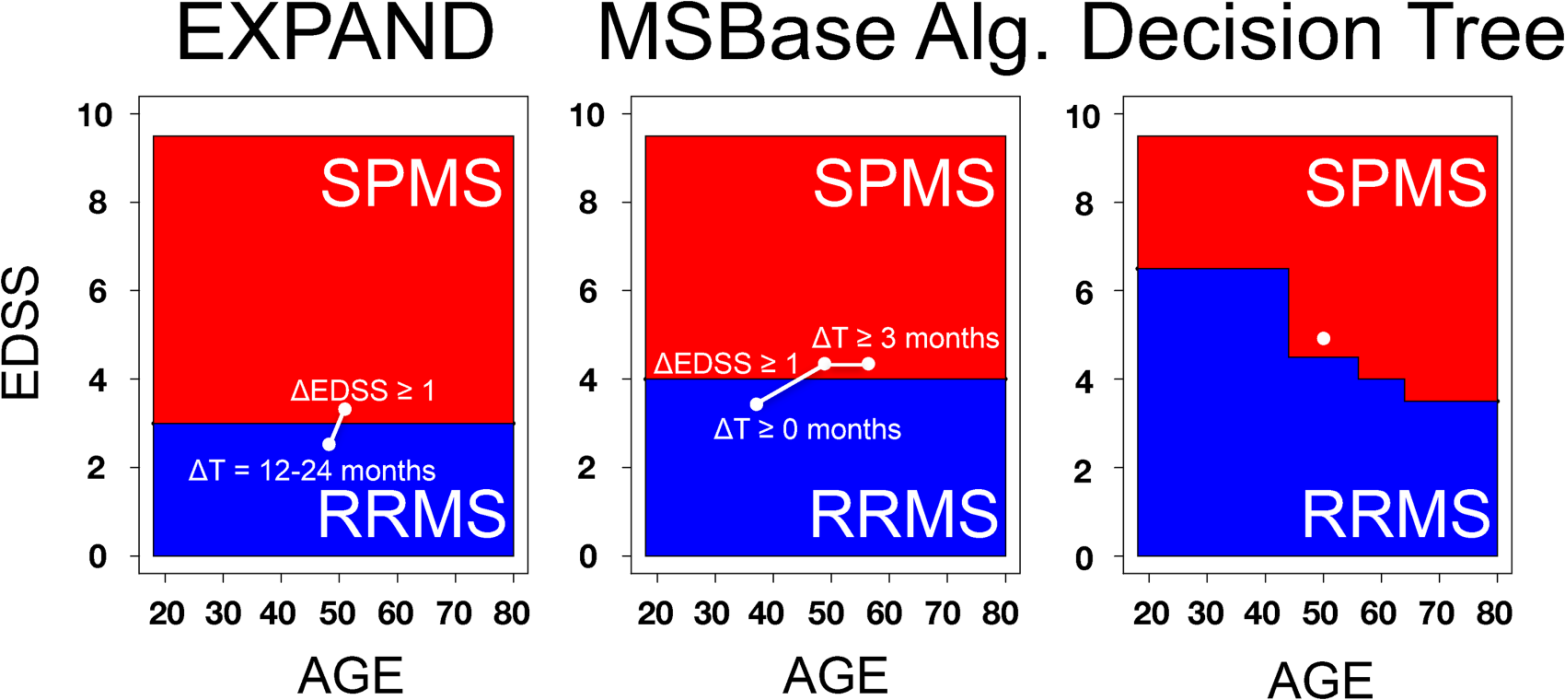
Comparison between methods for secondary progressive multiple sclerosis (SPMS) classification.

All three methods were implemented in a similar fashion. The algorithms scanned through each patient's historical EDSS observations from the first EDSS and onwards, using each visit as baseline and the following visits as follow-up (depending on the method). Each patient was labeled as unclassified before starting the classification methods. If a patient was first classified as RRMS and at a later observation reclassified as SPMS, the date of conversion was set to the first time point of SPMS classification. If a patient was classified to SPMS already from the first observation, a date of conversion could not be established but the patient would still be regarded as classifiable albeit without a conversion date. Figure 1 shows the differences between the methods.

### Federated analysis

A common data model (CDM) was defined to allow for each registry to extract the data and transform it into a common data format. After transforming the data to the common data format, each registry processed the data using a shared analysis script written in R^24^ that applied the different rules for the classification methods and created the aggregated tables used to create the results in this study. The R script, with a detailed description of the CDM, is available in the supplemental materials. In brief, the CDM contained four different tables; patients, visits, relapses, and DMTs. These tables contained information about date of birth, MS onset date, MS diagnosis date, year of SP conversion (if available), and longitudinal information about visits with EDSS observations, relapses, and DMTs.

### Concordance with clinical data

A concordance procedure was used to observe the level of agreement between each of the objective classification methods and the clinical assignment of RRMS/SPMS, where sensitivity, specificity, and accuracy were measured for each registry and across all registries, using the clinical assignment of course as the standard, in spite of its limitations. Sensitivity measured the proportion of clinically assigned SPMS patients being classified as SPMS by each method, specificity measured the proportion of clinically assigned RRMS patients being classified as RRMS by each method, and accuracy is the overall proportion of patients being objectively classified according to the clinical assignment.

## Results

### Proportion of SPMS

**Figure 2.**
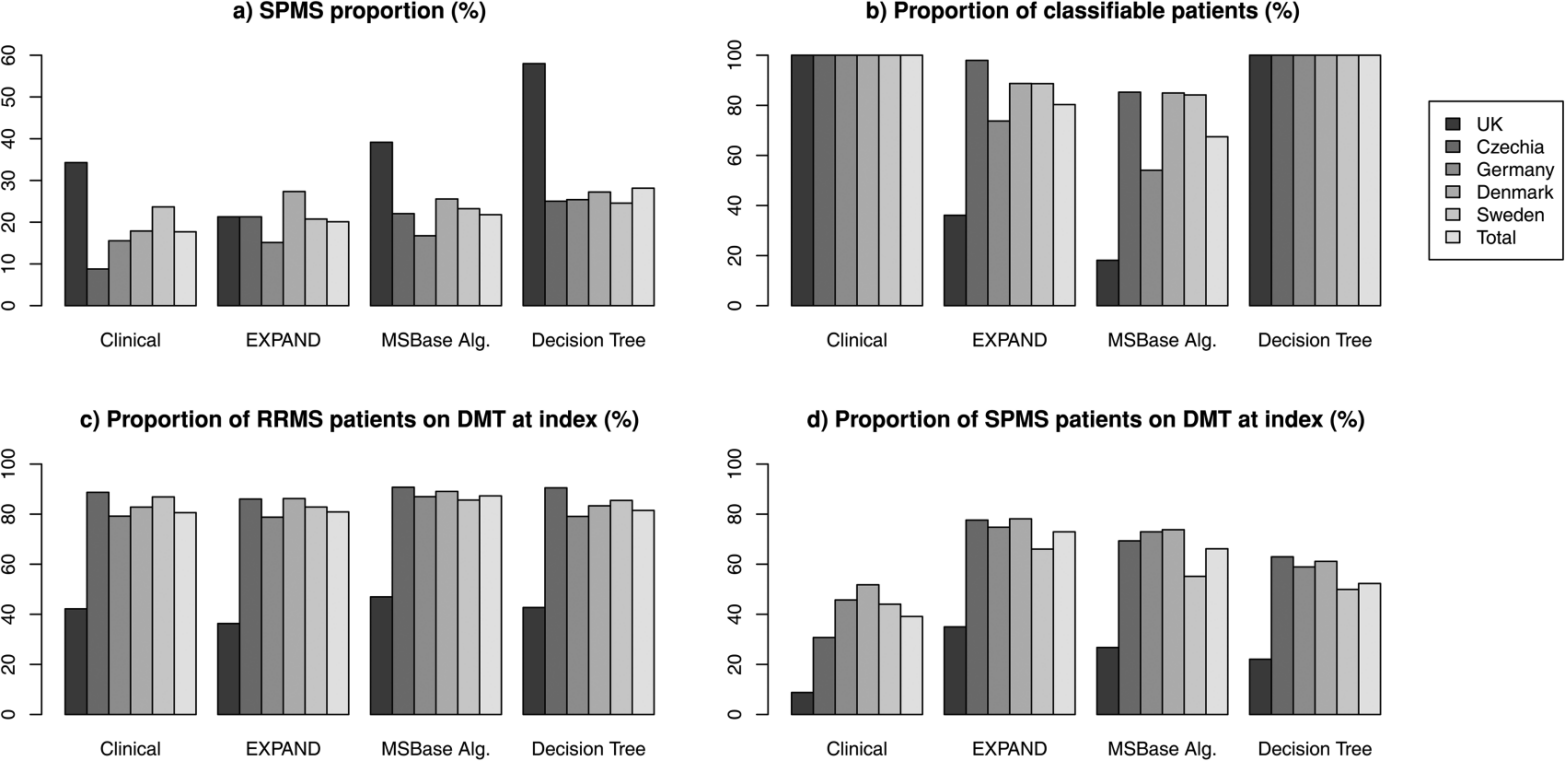
Proportions of patients between countries for each classifier. (a) Shows the SPMS proportion for each classifier among the classifiable patients. (b) Shows proportion of classifiable patients in the study population for each classifier. (c and d) Show proportion of RRMS and SPMS patients on DMT at index date for each classifier.

A total of 61,950 patients were included in this study. Of these 10,600 (17.7%) were clinically assigned an SPMS course, with a proportion that varied between the registries from 8.7% in Czechia to 34.3% in the UK. Application of each of the three objective classifiers increased the proportion in all registries as shown in Figure 2(a) for the different methods in each country and in total. Overall, the proportion of SPMS increased using objective classifiers: from 17.7% clinical assignment, 20.1% by EXPAND criteria, 21.8% by the MSBase algorithm, to 28.1% (17,402) according to the decision tree method.

The SPMS proportion differed substantially between countries but became more homogeneous with the objective methods compared to the clinical assignment. Not counting the UK register, which was an outlier in terms of proportion and population coverage, this was especially true for the decision tree method by which the SPMS assignment ranged between 24.6% and 27.2%, compared to from 8.8% to 23.7% for the clinical assignment.

It is important to note that the proportion of patients that could be classified by the different methods varied depending on their respective requirements of numbers of EDSS observations. Accordingly, the decision tree classifier, demanding only one EDSS, was able to classify all patients, whereas the EXPAND criteria and the MSBase algorithm classified 80% and 67%, respectively (Figure 2(b)). The average age of unclassifiable patients by each method was higher than the average age of RRMS patients and lower than the average age of SPMS patients for all methods (Table 3).

### Characteristics of SPMS

The SPMS groups defined by the objective classifiers differed from the clinically assigned SPMS group in terms of age, age at conversion, and median EDSS and EDSS at conversion.

The average age at index date and at SPMS conversion by each method is shown in Table 1. The average age at index date for RRMS patients was fairly consistent between the objective classifiers, ranging from 42.8 years (decision tree) to 45.2 years (EXPAND) across all registries. The average age at index date for SPMS patients ranged from 51.7 years (EXPAND) to 57.2 years (decision tree), demonstrating less consistency between the classifiers. Notably, patients assigned as SPMS by the decision tree methods were closest to the age of the clinically assigned SPMS patients at index date (57.2 years for decision tree, 57.9 years for clinical). The average age at SPMS conversion ranged from 46.6 years (EXPAND) to 50.5 years (decision tree). The decision tree was also in this case closest to the clinical age at conversion (49.1 years).

**Table 1.** Mean age (SD) at index date and at SPMS conversion for each method.

	UK	Czech Republic	Germany	Denmark	Sweden	Total
RRMS at index						
Clinical	47.0 (10.8)	43.7 (10.8)	44.2 (11.5)	45.5 (11.5)	43.8 (11.4)	44.4
EXPAND	52.7 (11.2)	43.9 (11.3)	45.0 (12.2)	45.2 (11.6)	45.5 (12.4)	45.2
MSBase alg.	49.7 (10.9)	41.8 (10.3)	43.5 (11.5)	45.2 (11.0)	45.0 (11.8)	43.9
Decision tree	43.8 (10.9)	41.1 (10.3)	42.6 (11.5)	43.9 (11.0)	43.6 (11.8)	42.8
SPMS at index						
Clinical	59.1 (8.7)	56.0 (10.4)	56.9 (9.9)	57.6 (10.1)	59.6 (10.7)	57.9
EXPAND	55.1 (8.7)	47.7 (10.4)	51.4 (9.9)	52.9 (10.1)	54.5 (10.7)	51.7
MSBase alg.	57.4 (9.7)	49.7 (10.5)	53.3 (10.7)	54.0 (10.2)	57.2 (11.3)	53.7
Decision tree	56.4 (9.6)	55.8 (8.8)	56.8 (9.3)	58.0 (9.8)	59.9 (10.6)	57.2
Unclassifiable at index						
EXPAND	50.0 (11.8)	49.2 (12.6)	46.7 (11.9)	51.2 (14.7)	49.3 (15.1)	48.3
MSBase alg.	50.8 (11.7)	51.9 (11.2)	47.4 (12.4)	49.4 (15.5)	46.2 (14.8)	48.5
SPMS conversion						
Clinical	50.0 (9.3)	46.2 (9.8)	49.0 (9.6)	50.8 (10.3)	48.7 (10.3)	49.1
EXPAND	52.7 (10.1)	44.4 (10.2)	48.1 (11.1)	46.5 (10.1)	47.7 (11.2)	46.6
MSBase alg.	54.1 (9.4)	44.3 (10.2)	48.1 (10.5)	45.4 (10.3)	48.7 (11.3)	46.7
Decision tree	51.6 (9.7)	49.1 (7.7)	51.3 (8.6)	49.6 (9.0)	51.7 (10.5)	50.5

RRMS: relapsing–remitting multiple sclerosis; SPMS: secondary progressive multiple sclerosis; UK: United Kingdom.

Table 2 shows the median EDSS at index date and at SPMS conversion which was fairly consistent between classifiers and also between registries, with the exception of the UK registry where patients were generally more disabled.

**Table 2.** Median (IQR) EDSS at index and at SPMS conversion for each method.

	UK	Czech Republic	Germany	Denmark	Sweden
RRMS at index					
Clinical	4.0 (2.5–6.0)	2.5 (1.5–4.0)	2.0 (1.0–3.5)	2.0 (1.0–3.0)	1.5 (1.0–2.5)
EXPAND	4.0 (2.0–6.5)	2.0 (1.5–3.5)	2.0 (1.0–3.5)	2.0 (1.0–2.5)	1.5 (1.0–2.5)
MSBase alg.	2.5 (2.0–3.0)	2.0 (1.5–3.0)	2.0 (1.0–2.5)	2.0 (1.0–2.5)	1.5 (1.0–2.5)
Decision tree	3.0 (2.0–4.0)	2.0 (1.5–3.0)	2.0 (1.0–3.0)	2.0 (1.0–2.5)	1.5 (1.0–2.5)
SPMS at index					
Clinical	6.5 (6.0–7.5)	6.5 (5.5–7.0)	6.0 (4.5–7.0)	6.0 (4.0–6.5)	6.0 (4.0–7.0)
EXPAND	6.5 (4.5–6.5)	4.5 (4.0–6.0)	5.5 (4.0–6.5)	5.5 (3.5–6.5)	5.5 (4.0–6.5)
MSBase alg.	6.5 (6.0–7.0)	5.5 (4.5–6.5)	6.0 (4.5–6.5)	6.0 (4.0–6.5)	6.0 (5.0–7.0)
Decision tree	6.5 (6.0–7.0)	5.5 (4.5–6.5)	6.0 (4.5–6.5)	6.0 (4.5–6.5)	6.0 (4.5–7.0)
Unclassifiable at index					
EXPAND	5.5 (3.5–6.5)	4.0 (3.0–5.5)	3.0 (2.0–4.5)	3.0 (2.0–5.5)	3.0 (1.5–4.5)
MSBase alg.	6.0 (3.5–6.5)	5.0 (4.0–5.5)	3.5 (2.0–5.5)	2.5 (1.5–5.0)	2.0 (1.0–4.5)
SPMS conversion					
Clinical	6.5 (6.0–7.0)	6.0 (4.5–6.5)	6.0 (4.0–6.5)	5.0 (3.5–6.5)	4.5 (3.0–6.0)
EXPAND	4.0 (3.5–5.0)	4.0 (3.5–5.5)	4.0 (3.5–5.5)	4.0 (3.5–5.0)	4.0 (3.5–5.0)
MSBase alg.	4.0 (4.0–4.4)	4.0 (4.0–4.5)	4.0 (4.0–5.0)	4.5 (4.0–5.5)	4.5 (4.0–5.5)
Decision tree	5.0 (4.0–6.5)	4.5 (4.5–5.5)	5.0 (4.5–6.0)	5.5 (4.5–6.5)	5.0 (4.5–6.0)

EDSS: Expanded Disability Status Scale; RRMS: relapsing–remitting multiple sclerosis; SPMS: secondary progressive multiple sclerosis; UK: United Kingdom.

The proportion of SPMS patients with relapses within the last 2 years was in all cases higher among objectively classified SPMS patients compared to those clinically assigned SPMS ranging from 8.0% for clinically assigned SPMS to 22.4% for EXPAND (Table 3). The proportion of RRMS patients with relapses was similar between the different methods.

**Table 3.** Proportion (%) of patients with relapses during a time window of 2 years prior to index date.

	UK	Czech Republic	Germany	Denmark	Sweden	Total
RRMS						
Clinical	8.0	38.6	16.8	17.1	9.6	19.5
EXPAND	9.8	33.9	15.9	15.3	8.5	18.1
MSBase alg.	9.9	37.0	18.4	16.3	8.0	19.8
Decision tree	7.2	39.1	16.4	16.6	9.6	19.0
SPMS						
Clinical	5.8	14.8	9.3	9.8	3.6	8.0
EXPAND	12.1	42.7	22.3	19.0	5.4	22.4
MSBase alg.	8.9	35.1	18.5	17.5	4.2	18.3
Decision tree	7.3	28.6	13.4	13.7	4.1	13.4
Unclassifiable						
EXPAND	5.5	70.9	12.2	11.7	10.9	11.3
Decision tree	6.7	35.9	12.3	11.3	14.0	13.2

RRMS: relapsing–remitting multiple sclerosis; SPMS: secondary progressive multiple sclerosis; UK: United Kingdom.

### Proportion of DMT use

As shown in Figure 2, the proportion of SPMS patients on DMTs were in all cases higher among objectively classified SPMS patients compared to those clinically assigned SPMS, ranging from 39.1% by clinical assignment to 72.9% by the EXPAND classifier. The proportion of RRMS on DMT treatment differed less between clinical assignment and the objective classifiers.

### Comparison of classifiers

The ability to classify clinical SPMS patients as SPMS, measured as the *sensitivity* of the methods, was quite different between the methods (47.4% EXPAND, 75.7% for MSBase algorithm, and 83.5% for decision tree). The ability to classify clinical RRMS patients as RRMS, measured as *specificity*, was more consistent between the methods (85.1% EXPAND, 87.0% MSBase algorithm, and 83.7% decision tree). The overall accuracy of each method against the clinical assignment was 79.1% for EXPAND, 85.4% for the MSBase algorithm, and 83.7% for the decision tree classifier. A detailed summary of these results is shown in Supplemental Table S1.

## Discussion

We report that objective classifiers of RRMS/SPMS systematically assign a higher proportion of SPMS in registry-based MS populations, suggesting a systematic underestimation of SPMS and indicating that many MS patients clinically assigned an RRMS course are likely to have clinical features compatible with an SPMS course.

We applied three objective classifiers on data from five European MS registries and compared them with clinically assigned courses. In contrast to clinical criteria, objective classifiers are confined to identifying SPMS merely on EDSS to determine the likelihood of SPMS, since gradual progression, the hallmark of SPMS, is not an independent item in MS registries. This limitation does not preclude that algorithms may reach a high degree of precision at the group level, as shown by validation analyses.^14,18^

Most strikingly, our results suggest that the SPMS population may be considerably larger than indicated by clinical MS registries, even among those with a high population coverage, where the true proportion appears to be close to 30%, rather than less than 20%. This is in good accordance with the classic proportion of 27% SPMS in the Lyon natural history cohort^13^ and other population-based estimations.^25^

Importantly, we find that the algorithms propose reclassification more often among patients that are younger, female, have more relapses, and are on DMTs. Since young age, female sex, and relapses are characteristics of RRMS, it should be expected that such events tend to delay a correct assignment of conversion to SPMS, but can alternatively reflect less than optimal specificity of the objective classifiers. However, it is less likely that patients on DMTs are unconsciously misclassified by clinicians because they are on treatment. Thus, it is tempting to speculate that the lack of treatment options for SPMS (until recently) have rendered neurologists less prone to assign patients to a disease course label which would make them ineligible for DMTs. The consistency of this observation between contributing registries indicates that this is a general phenomenon and less sensitive to specific administrative requirements surrounding the prescription of DMTs in different countries.

We used three different objective definitions of SPMS, classifiers, which objectively allocate a patient to either an RRMS or SPMS course based on likelihood estimations and principally based on EDSS instead of on reported gradual progression. In general, each classifier had a high level of accordance with the clinical assignment, which is shown by the high sensitivity and specificity scores obtained. Therefore, the classifiers performed similarly as the clinical assignments for most patients. However, the classifiers allowed us to harmonize the SPMS assignments between registries according to an objective standard. In that regard, the decision tree classifier was superior since it only required information on age and one single EDSS score, and was therefore able to classify all included patients, which can be expressed as a superior sensitivity at the cost of a modestly lower specificity.

A strength of this study is its size, with an analysis based on over 60,000 MS patients of which 10,000 patients were assigned SPMS clinically and over 17,000 SPMS patients according to the decision tree method, making it a uniquely large SMPS study. Another strength is the consistency between four of the five registries, whereas the UK registry clearly seems to be biased toward an older and more advanced MS population. Whereas the Czech, Danish and Swedish registries all have over 80% coverage of their respective populations and thus being quite representative, the estimated population coverage of the UK MS registry is a mere 13%.

There are clearly inherent challenges in applying criteria on something as poorly defined as the conversion from RRMS to SPMS. The most fundamental aspect is evidently whether or not RRMS and SPMS are meaningful entities. Due to evidence of progressive CNS tissue loss in RRMS and relapses well into the SPMS stage, some authors have even proposed that a proper description of MS course is a continuum rather than staged.^21^ On the other hand, the widely embraced concept that relapses and progression require different treatment approaches, importantly underlying all levels of MS drug development including labels of DMTs, argues for a remaining operational if not biological relevance of a two-staged perception of MS regardless of its imprecise nature. For a review of the strengths and weaknesses of the RRMS/SPMS dichotomy, please see Cree et al.^10^

Another challenge is also the starting point of this study, that is, a perceived weakness of the clinical assignment of SPMS, which is indeed supported and further underscored by our results. Thus, the objective EDSS-based classifiers lack a “gold standard” to be compared with. Since the used classifiers by definition assign the most likely course to a given patient based on EDSS, there will be patients with a clinical progression being classified as RRMS and patients without progression in the SPMS group. Therefore, and since clinicians will have access to the patient's history of progression or not, a classifier is not intended to be used in clinics or instead of clinical information but only when such information is lacking, that is, when dealing with patient cohorts in registries or other databases.

It may seem as a weakness that features of RRMS, such as female sex and relapse activity are more frequent in patients reclassified to SPMS than in clinically assigned SPMS as this would be the result of a poor performance of the classifiers which would be expected to overestimate the proportion of SPMS. However, due to the design of the classifiers, older patients with an early progression may be misclassified as RRMS, which to an unknown extent will counteract an overestimation of the SPMS proportion.

The third limitation is the difficulty to identify at which time point, that is, at what age or disease duration, that transition from RRMS to SPMS occurs. Clearly, the transition from one poorly defined stage of disease into another cannot be expected to be more than an approximation but when compared at the group level may still be informative. Clinically, the time of transition is retrospectively identified looking back at what approximate time a gradual progression was first experienced, that is, a task potentially sensitive to bias. In contrast, objective EDSS-based algorithms by definition may appear more robust but are restricted to time points when a progression is confirmed as a higher EDSS score. Thus, algorithms define the time of conversion with some delay, evidently sensitive to such factors as visit frequency and limitations of the EDSS scale. Further development of these classifiers to adjust for this inherent delay is warranted.

The decision tree method has the obvious advantage over the MSBase and modified EXPAND classifiers of requiring only one EDSS, but this study was not intended to make a formal comparison of other properties between these approaches.

Overall, we have reasons to believe that the objective classifiers are correct in showing a higher proportion of SPMS in the studied databases and registries: First, there is an à priori suspicion that clinicians have reasons to deviate by avoiding to identify an SPMS course not to render patients untreatable. Second, all classifiers behave similarly by increasing the proportion of SPMS. Third, the resulting proportions are more similar to relevant previously published population-based studies.

Finally, these findings may be of relevance for our understanding of the MS disease course and for design and planning of MS care services. In addition, it is questionable whether epidemiological studies based on MS registry data should use conversion to SPMS as an outcome measure. Use of EDSS milestones, such as EDSS 3, 4, or 6, or confirmed disability progression, seem to be better choices.

In conclusion, the application of objective MS course classifiers when compared with the clinical assignment of SPMS shows that the proportion of SPMS may be consistently underestimated in clinical MS registries and databases. Thus, many MS patients clinically assigned as RRMS are reclassified as SPMS when objective disease course classifiers are applied. Such patients are more commonly on DMTs compared to patients clinically assigned as SPMS. Thus, the population of SPMS eligible for new DMTs labeled for SPMS may in several populations be larger than expected.

## Supplemental Material

sj-docx-1-mso-10.1177_20552173231153557 - Supplemental material for Proportion and characteristics of secondary progressive multiple sclerosis in five European registries using objective classifiers Click here for additional data file. Supplemental material, sj-docx-1-mso-10.1177_20552173231153557 for Proportion and characteristics of secondary progressive multiple sclerosis in five European registries using objective classifiers by Lars Forsberg, Tim Spelman, Pernilla Klyve, Ali Manouchehrinia, Ryan Ramanujam, Elena Mouresan, Jiri Drahota, Dana Horakova, Hanna Joensen, Luigi Pontieri, Melinda Magyari, David Ellenberger, Alexander Stahmann, Jeff Rodgers, James Witts, Rod Middleton, Richard Nicholas, Vladimir Bezlyak, Nicholas Adlard, Thomas Hach, Carol Lines, Sandra Vukusic, Merja Soilu-Hänninen, Anneke van der Walt, Helmut Butzkueven, Pietro Iaffaldano, Maria Trojano, Anna Glaser, Jan Hillert and in Multiple Sclerosis Journal – Experimental, Translational and Clinical

sj-pdf-2-mso-10.1177_20552173231153557 - Supplemental material for Proportion and characteristics of secondary progressive multiple sclerosis in five European registries using objective classifiers Click here for additional data file. Supplemental material, sj-pdf-2-mso-10.1177_20552173231153557 for Proportion and characteristics of secondary progressive multiple sclerosis in five European registries using objective classifiers by Lars Forsberg, Tim Spelman, Pernilla Klyve, Ali Manouchehrinia, Ryan Ramanujam, Elena Mouresan, Jiri Drahota, Dana Horakova, Hanna Joensen, Luigi Pontieri, Melinda Magyari, David Ellenberger, Alexander Stahmann, Jeff Rodgers, James Witts, Rod Middleton, Richard Nicholas, Vladimir Bezlyak, Nicholas Adlard, Thomas Hach, Carol Lines, Sandra Vukusic, Merja Soilu-Hänninen, Anneke van der Walt, Helmut Butzkueven, Pietro Iaffaldano, Maria Trojano, Anna Glaser, Jan Hillert and in Multiple Sclerosis Journal – Experimental, Translational and Clinical
