# Supplementary material for: Proportion and characteristics of secondary progressive multiple sclerosis in five European registries using objective classifiers
Source: Mult Scler J Exp Transl Clin. 2023 Feb 16;9(1):20552173231153557. doi: 10.1177/20552173231153557 (PMC9936396; doi:10.1177/20552173231153557)
Supplement: sj-docx-1-mso-10.1177_20552173231153557 - Supplemental material for Proportion and characteristics of secondary progressive multiple sclerosis in five European registries using objective classifiers [file sj-docx-1-mso-10.1177_20552173231153557.docx]

Supplement table 1: Concordance (%) between classification methods and the clinical assignment.

|  | UK | Czech Republic | Germany | Denmark | Sweden | Total |
| --- | --- | --- | --- | --- | --- | --- |
| **EXPAND** |  |  |  |  |  |  |
| Sensitivity | 26.4 | 41.2 | 36.3 | 67.1 | 55.2 | 47.4 |
| Specificity | 81.5 | 80.6 | 88.4 | 80.5 | 89.4 | 85.1 |
| Accuracy | 62.1 | 77.2 | 80.9 | 78.3 | 81.6 | 79.1 |
| **MSBase Alg.** |  |  |  |  |  |  |
| Sensitivity | 87.0 | 91.9 | 73.6 | 71.9 | 73.9 | 75.7 |
| Specificity | 76.9 | 82.5 | 89.8 | 83.8 | 92.5 | 87.0 |
| Accuracy | 79.4 | 83.1 | 88.1 | 81.8 | 88.1 | 85.4 |
| **Decision Tree** |  |  |  |  |  |  |
| Sensitivity | 95.0 | 88.3 | 82.7 | 77.8 | 79.4 | 83.5 |
| Specificity | 61.3 | 81.1 | 85.2 | 83.8 | 92.5 | 83.8 |
| Accuracy | 72.9 | 81.7 | 84.8 | 82.7 | 89.4 | 83.7 |
