# Supplementary material for: Proportion and characteristics of secondary progressive multiple sclerosis in five European registries using objective classifiers
Source: Mult Scler J Exp Transl Clin. 2023 Feb 16;9(1):20552173231153557. doi: 10.1177/20552173231153557 (PMC9936396; doi:10.1177/20552173231153557)
Supplement: sj-pdf-2-mso-10.1177_20552173231153557 - Supplemental material for Proportion and characteristics of secondary progressive multiple sclerosis in five European registries using objective classifiers [file sj-pdf-2-mso-10.1177_20552173231153557.pdf]

```r
# This is the analysis script written in R for the article
# "Proportion and characteristics of secondary progressive multiple
# sclerosis in five European registries using objective classifiers"
#
# This script assumes the following data frames and with the
# following columns:
#  - patients
#     - patientcode     (a unique number for each patient)
#     - dateofbirth     (text string in the format YYYY-MM-DD)
#     - gender          (either "M" or "F")
#     - onsetdate       (text string in the format YYYY-MM-DD,
#                       NA if unknown)
#     - diagnosis date  (text string in the format YYYY-MM-DD,
#                       NA if unknown)
#     - mstype          (either "RR" or "SP")
#     - spyear          (year of clinical SP-conversion,
#                       NA if unknown or not applicable)
#  - visits
#     - patientcode     (a unique number for each patient)
#     - date            (date for each visit in the format
#                       YYYY-MM-DD)
#     - edss            (EDSS observation at the visit -
#                       mandatory)
#     - pyramidalfss    (pyramidal functional system score,
#                       NA if unknown)
#  - relapses
#     - patientcode     (a unique number for each patient)
#     - date            (date of relapse)
#  - dmt
#     - patientcode     (a unique number for each patient)
#     - startdate       (start date of the treatment as text
#                       string in the format YYYY-MM-DD, mandatory)
#     - stopdate        (stop date of the treatment as text string
#                       in the format YYYY-MM-DD, NA if ongoing)
#     - name            (name of DMT)
#
# The DMT names are assumed to be one of the following:
#
# "Alemtuzumab","Azathioprine","Beta-Interferon","Cladribine",
# "Clinical Trial", "Daclizumab","Dimethyl Fumarate","Fingolimod",
# "Glatiramer Acetate","Immunoglobulin", "Methotrexate","Mitoxantrone",
# "Natalizumab","Ocrelizumab","Ofatumumab","Plasmapheresis", "Rituximab",
# "Siponimod","Stem Cell Treatment","Teriflunomide","Methylprednisolone",
# "Treatment Not Specified"

version <- 1.9

# Set date types - this will speed things up when making date comparisons
patients$dateofbirth <- as.Date(patients$dateofbirth)
patients$onsetdate <- as.Date(patients$onsetdate)
patients$diagnosisdate <- as.Date(patients$diagnosisdate)
visits$date <- as.Date(visits$date)
relapses$date <- as.Date(relapses$date)
```

```
dmt$startdate <- as.Date(dmt$startdate)
dmt$stopdate <- as.Date(dmt$stopdate)

dmtnames <- c("Alemtuzumab","Azathioprine","Beta-Interferon","Cladribine","Clinical Tri
Fumarate","Fingolimod","Glatiramer
Acetate","Immunoglobulin","Methotrexate","Mitoxantrone","Natalizumab","Ocrelizumab","Of
Cell Treatment","Teriflunomide","Methylprednisolone","Treatment Not Specified")



# Quality Check for undefined names
if(nrow(subset(dmt,!dmt$name %in% dmtnames))>0) {
  print("DMT data has undefined DMT names:")
  print(unique(subset(dmt,!dmt$name %in% dmtnames)$name))
  stop("Stopping script due to undefined DMT names.")
}


print(sprintf("Total number of patients: %d",nrow(patients)))

# Inclusion/exclusion - include only patients having a visit with EDSS
between 2017-2019 (three years).
# Also remove visits in 2020 if any. Only patients with known birth date
are included.
visits <- subset(visits,is.na(visits$edss)==FALSE)
visits <- subset(visits,visits$date<as.Date("2020-01-01"))
visits2 <- subset(visits,visits$date>=as.Date("2017-01-01") &
as.Date(visits$date)<"2020-01-01")
patients <- subset(patients,patients$patientcode %in% visits2$patientcode)
patients <- subset(patients,is.na(patients$dateofbirth)==FALSE)
rm(visits2)

visitsFS <- subset(visits,is.na(visits$pyramidalfss)==FALSE)

# Some data cleaning
## Set SP year to NA if the year > 2020 (probably some error)
if(sum(patients$spyear>2020,na.rm=TRUE)>0) {
  patients[is.na(patients$spyear)==FALSE & patients$spyear>2020,]$spyear <-
NA
}
## Set SP year to NA and mstype to RR if the year = 2020
if(sum(patients$spyear==2020,na.rm=TRUE)>0) {
  patients[is.na(patients$spyear)==FALSE & patients$spyear==2020,]$spyear
<- NA
}

## Set SP year to NA if SP year < MS onset date
if(sum(is.na(patients$spyear)==FALSE & is.na(patients$onsetdate)==FALSE &
as.Date(sprintf("%s-12-31",patients$spyear),"%Y-%m-
%d")<patients$onsetdate)>0)
```

```
  patients[is.na(patients$spyear)==FALSE & is.na(patients$onsetdate)==FALSE
& as.Date(sprintf("%s-12-31",patients$spyear),"%Y-%m-
%d")<patients$onsetdate,]$spyear <- NA

print(sprintf("Number of patients with index date and birth date:
%d",nrow(patients)))

patients$indexdate <- as.Date("1900-01-01")

print("Getting index date for each patient. This will take some while.")

# Get index date for each patient. This date is defined as the latest date
with an
# EDSS observation between 2017 to 2019.
patients$indexedss <- as.numeric(NA)

patients$indexedsscounts <- 0
patients$dmtatindex <- FALSE
patients$dmtatconversion <- FALSE
patients$dmtnameatindex <- ""
patients$indexrelapses <- 0
for(row in 1:nrow(patients)) {
  if(row %% 100 == 0)
    print(sprintf("Patients: %d",row))
  patient_visits <- subset(visits,visits$patientcode == patients[row,]
$patientcode)
  patient_visits <- patient_visits[rev(order(patient_visits$date)),]
  indexdate <- patient_visits$date[1]
  patients[row,]$indexedsscounts <-
sum(patient_visits$date>=(indexdate-365.25*5) &
patient_visits$date<=indexdate)
  patients[row,]$indexdate <- indexdate
  patients[row,]$indexedss <- patient_visits$edss[1]

  patient_dmts <- subset(dmt,dmt$patientcode == patients[row,]$patientcode)
  # If we find start date before index date and no stop date, this DMT is
used on index date
  # If we find start date before index date and stop date is 12 months
before index, we include this DMT as well
  patient_dmts <- subset(patient_dmts,patient_dmts$startdate<=indexdate &
(is.na(patient_dmts$stopdate)==TRUE |
patient_dmts$stopdate>=(indexdate-365)))
  if(nrow(patient_dmts)>0) {
    patients[row,]$dmtatindex <- TRUE
    # Select the DMT with the start date closest to index date
    patient_dmts <- patient_dmts[rev(order(patient_dmts$startdate)),]
    patients[row,]$dmtnameatindex <- patient_dmts[1,]$name
  }

  # Get number of relapses <=2 years prior to index date for this patient
  patient_relapses <- subset(relapses,relapses$patientcode==patients[row,]
$patientcode)
```

```
  patient_relapses <-
subset(patient_relapses,patient_relapses$date<=indexdate &
patient_relapses$date>=(indexdate-365*2))
  patients[row,]$indexrelapses <- nrow(patient_relapses)
}
patients$ageatindex <- as.numeric(patients$indexdate-patients$dateofbirth)/
365.25
patients <- subset(patients,patients$ageatindex>=18)

print(sprintf("Number of patients with age>=18 at index:
%d",nrow(patients)))

visits <- subset(visits,visits$patientcode %in% patients$patientcode)
relapses <- subset(relapses,relapses$patientcode %in% patients$patientcode)
dmt <- subset(dmt,dmt$patientcode %in% patients$patientcode)


# Get clinical EDSS at conversion ± 12 months
if(sum(is.na(patients$mstype)==TRUE)>0)
  patients[is.na(patients$mstype),]$mstype <- ""
print(sprintf("Getting clinical convertion date and EDSS around time of
conversion"))
patients$clinical_edss <- NA
patients$clinicalmstype <- patients$mstype
patients$clinicalspdate <- as.Date(sprintf("%s-07-02",patients$spyear),"%Y-
%m-%d")

for(row in 1:nrow(patients)) {
  if(row %% 100 == 0)
    print(sprintf("Patients: %d",row))
  patient <- patients[row,]

  if(is.na(patient$spyear)==TRUE)
    next
  patient_visits <- subset(visits,visits$patientcode ==
patient$patientcode)
  patient_visits$timesincesp <- abs(as.numeric(patient_visits$date-
as.Date(sprintf("%s-07-02",patient$spyear)))/365)
  patient_visits <- patient_visits[order(patient_visits$timesincesp),]
  patients[row,]$clinical_edss <- patient_visits[1,]$edss
  if(patients[row,]$mstype=="SP" && patients[row,]
$clinicalspdate>patients[row,]$indexdate) {
    patients[row,]$mstype=="RR"
  }
}

expand <- function(patient,debug) {
  patient_visits <- subset(visits,visits$patientcode == patient)
  patient_visits <- patient_visits[order(patient_visits$date),]

  patient_relapses <- subset(relapses,relapses$patientcode == patient)
  patient_relapses <- patient_relapses[order(patient_relapses$date),]
```

```
  if(debug==TRUE) {
    for(i in 1:nrow(patient_visits))
      print(sprintf("Date: %s. EDSS: %f",patient_visits[i,]
$date,patient_visits[i,]$edss))
  }

  if(nrow(patient_visits)<2) {
    if(debug==TRUE)
      print(sprintf("Not enough visits: %d",nrow(patient_visits)))
    return()
  }

  expand <- ""
  expandSPdate <- as.Date(NA)
  expand_edss <- NA

  for(i in 1:(nrow(patient_visits)-1)) {
    if(expand == "SP") # Already classified to SP
      next

    baseline <- patient_visits[i,]
    followup <- patient_visits[i+1,]

    # If any future EDSS<3.0, this patient is currently RR
    # and any EDSS>=3.0 in between are false SP.
    for(j in (i+1):nrow(patient_visits)) {
      if(patient_visits[j,]$edss<3.0) {
        expand <- "FutureRR"
      }
    }
    if(expand == "FutureRR") {
      if(debug==TRUE)
        print(sprintf("Future EDSS<3! Skipping. Followup date was: %s. EDSS
= %f  -> RR",followup$date,followup$edss))

      expand <- "RR"
      next
    }

    j <- 1
    while((followup$date-baseline$date)<365) { # Followup too close in
time, find next
      if(i+j>nrow(patient_visits))
        break
      followup <- patient_visits[i+j,]
      j <- j + 1
    }

    if((followup$date-baseline$date)<365) {
      # It must be at least 12 months between baseline and followup.
      next
    }
```

```
    # If baseline EDSS<3.0, patient is RR at that time point.
    if(baseline$edss<3.0) {
      if(debug==TRUE)
        print(sprintf("Baseline EDSS<3. Followup date: %s. EDSS = %f  ->
RR",followup$date,followup$edss))
      expand <- "RR"
    }

    # If followup EDSS<3.0, patient is always RR regardless of
    # time distance from baseline to followup.
    if(followup$edss<3.0) {
      expand <- "RR"
      if(debug==TRUE)
        print(sprintf("Followup date: %s. EDSS = %f  ->
RR",followup$date,followup$edss))
      next
    }

    # At this point, patient has EDSS>=3.0 and may have converted to SP.

    if((followup$date-baseline$date)>365*2) {
      # Too large gap between baseline and followup to
      # determine. Patient is unclassifiable for now.
      expand <- ""
      if(debug==TRUE)
        print(sprintf("GAP. Followup date: %s. EDSS = %f  ->
NONE",followup$date,followup$edss))
      next
    }

    if(followup$edss<6.0) {
      if(followup$edss-baseline$edss>=1) {
        if(expand == "RR") { # Only set SP date if previous expand was RR
          if(debug==TRUE)
            print("SET SP DATE")
          expandSPdate <- followup$date
          expand_edss <- followup$edss
        }
        if(debug==TRUE)
          print(sprintf("Currently: %s. SP convert. Baseline date %s. EDSS %f.
Followup date: %s. EDSS = %f  -> SP. Conversion date:
%s",expand,baseline$date,baseline$edss,followup$date,followup$edss,expandSPdate))
        expand <- "SP"
        break
      }
      if(followup$edss-baseline$edss<1 && expand != "SP") {
        expand <- "RR" # Only set SP date if previous expand was RR
        if(debug==TRUE)
          print(sprintf("Followup date: %s. EDSS = %f  ->
RR",followup$date,followup$edss))
      }
    }
    if(followup$edss>=6.0) {
```

```
      if(followup$edss-baseline$edss>=0.5) {
        if(expand == "RR") {
          expandSPdate <- followup$date
          expand_edss <- followup$edss
        }
        expand <- "SP"
        if(debug==TRUE)
          print(sprintf("Followup date: %s. EDSS = %f  -> SP. Conversion
date:",followup$date,followup$edss,expandSPdate))
        break
      }
      if(followup$edss-baseline$edss<0.5 && expand != "SP") {
        expand <- "RR"
        if(debug==TRUE)
          print(sprintf("Followup date: %s. EDSS = %f  ->
RR",followup$date,followup$edss))
        break
      }
    }
  }
  patients[patients$patientcode==patient,]$expand_mstype <<- expand
  patients[patients$patientcode==patient,]$expand_spdate <<- expandSPdate
  patients[patients$patientcode==patient,]$expand_edss <<- expand_edss
  if(debug==TRUE)
    print(sprintf("Final result: EXPAND MSTYPE = %s. EXPAND SP DATE = %s.
EXPAND EDSS= %f",expand,expandSPdate,expand_edss))
}

# Get EXPAND
patients$expand_mstype <- ""
patients$expand_spdate <- as.Date(NA)
patients$expand_edss <- NA

print(sprintf("Getting EXPAND classification"))

for(row in 1:nrow(patients)) {
  if(row %% 100 == 0)
    print(sprintf("Patients: %d",row))
  patient <- patients[row,]
  expand(patient$patientcode,FALSE)
}

# MSBase algorithm function
msbaseAlgorithm <- function(patient,debug) {
  patient_visits <- subset(visits,visits$patientcode == patient)
  patient_visits <- patient_visits[order(patient_visits$date),]

  msbasealgorithm_mstype <- ""
  msbasealgorithm_spdate <- as.Date(NA)
  msbasealgorithm_edss <- as.numeric(NA)

  patient_relapses <- subset(relapses,relapses$patientcode == patient)
  patient_relapses <- patient_relapses[order(patient_relapses$date),]
```

```
  if(nrow(patient_visits)<3)
    return()

  for(i in 1:(nrow(patient_visits)-2)) {
    baseline <- patient_visits[i,]
    if(msbasealgorithm_mstype=="SP")
      next
    for(j in (i+1):(nrow(patient_visits)-1)) {
      if(msbasealgorithm_mstype=="SP")
        next
      followup <- patient_visits[j,]

      if(baseline$edss<4)
        msbasealgorithm_mstype <- "RR"
      if(followup$edss<4) {
        msbasealgorithm_mstype <- "RR"
        next
      }

      relapse_activity <-
subset(patient_relapses,patient_relapses$date>=followup$date-30 &
patient_relapses$date<=followup$date)
      if(nrow(relapse_activity)>0)
        next

      confirmed <- FALSE
      if(followup$edss<=5.5) {
        if(followup$edss-baseline$edss>=1.0) {
          # We may have a convert, but need to confirm with a confirmation
EDSS >=3 months
          for(k in (j+1):nrow(patient_visits)) {
            confirm <- patient_visits[k,]
            if(confirm$date-followup$date>=90) {
              if(confirm$edss>=followup$edss) {
                relapse_activity <-
subset(patient_relapses,patient_relapses$date>=confirm$date-30 &
patient_relapses$date<=confirm$date)
                if(nrow(relapse_activity)==0)
                  confirmed <- TRUE
              }
              break
            }
          }
        }
      }
      if(followup$edss>=6.0) {
        if(followup$edss-baseline$edss>=0.5) {
          # We may have a convert, but need to confirm with a confirmation
EDSS >=3 months
          confirmed <- FALSE
          for(k in (j+1):nrow(patient_visits)) {
            confirm <- patient_visits[k,]
```

```
            if(confirm$date-followup$date>=90) {
              if(confirm$edss>=followup$edss) {
                relapse_activity <-
subset(patient_relapses,patient_relapses$date>=confirm$date-30 &
patient_relapses$date<=confirm$date)
                if(nrow(relapse_activity)==0)
                  confirmed <- TRUE
              }
              break
            }
          }
        }
      }

      if(confirmed==TRUE) {
        if(msbasealgorithm_mstype=="RR") {
          msbasealgorithm_spdate <- followup$date
          msbasealgorithm_edss <- followup$edss
        }
        msbasealgorithm_mstype <- "SP"
      }
    }
  }
  patients[patients$patientcode==patient,]$msbasealgorithm_mstype <<-
msbasealgorithm_mstype
  patients[patients$patientcode==patient,]$msbasealgorithm_spdate <<-
msbasealgorithm_spdate
  patients[patients$patientcode==patient,]$msbasealgorithm_edss <<-
msbasealgorithm_edss
}

# Get msbasealgorithm
patients$msbasealgorithm_mstype <- ""
patients$msbasealgorithm_spdate <- as.Date(NA)
patients$msbasealgorithm_edss <- as.numeric(NA)
print(sprintf("Getting MSBase classification"))
for(row in 1:nrow(patients)) {
  if(row %% 100 == 0)
    print(sprintf("Patients: %d",row))
  patient <- patients[row,]
  msbaseAlgorithm(patient$patientcode,FALSE)
}

# MSBase algorithm function with FS score
msbaseAlgorithmFS <- function(patient,debug) {
  patient_visits <- subset(visits,visits$patientcode == patient)
  patient_visits <- patient_visits[order(patient_visits$date),]

  msbasealgorithm_mstype <- ""
  msbasealgorithm_spdate <- as.Date(NA)
  msbasealgorithm_edss <- NA

  patient_relapses <- subset(relapses,relapses$patientcode == patient)
```

```
  patient_relapses <- patient_relapses[order(patient_relapses$date),]

  if(nrow(patient_visits)<3)
    return()

  for(i in 1:(nrow(patient_visits)-2)) {
    baseline <- patient_visits[i,]
    if(msbasealgorithm_mstype=="SP")
      next
    for(j in (i+1):(nrow(patient_visits)-1)) {
      if(msbasealgorithm_mstype=="SP")
        next
      followup <- patient_visits[j,]

      if(baseline$edss<4)
        msbasealgorithm_mstype <- "RR"
      if(followup$edss<4) {
        msbasealgorithm_mstype <- "RR"
        next
      }

      # Followup is EDSS≥4
      # Followup must have pyramidal FSS and it must be >=2.
      if(is.na(followup$pyramidalfss)==TRUE &&
msbasealgorithm_mstype=="RR") {
        msbasealgorithm_mstype <- "RR2" # RR2 = RR patient that will be
unclassified if a future visit does not convert patient to SPMS.
        next # Go to next visit.
      }
      if(msbasealgorithm_mstype=="RR2")
        msbasealgorithm_mstype <- "RR" # We found another visit with FS-
score. Set back to RR if RR2.
      if(is.na(followup$pyramidalfss)==TRUE || followup$pyramidalfss<2)
        next # Not SPMS

      # At this point, followup is both EDSS>=4 and pyramidal FSS >= 2.

      relapse_activity <-
subset(patient_relapses,patient_relapses$date>=followup$date-30 &
patient_relapses$date<=followup$date)
      if(nrow(relapse_activity)>0) {
        # Here, we could set patient to RR2 because of uncertainty.
        # For now, it will remain RRMS, with the notion that
        # this patient is getting increased
        next
      }

      confirmed <- FALSE
      if(followup$edss<=5.5) {
        if(followup$edss-baseline$edss>=1.0) {
          # We may have a convert, but need to confirm with a confirmation
EDSS >=3 months
          for(k in (j+1):nrow(patient_visits)) {
```

```
            confirm <- patient_visits[k,]
            # Get first EDSS & FSS >= 3 months
            if(confirm$date-followup$date>=90 &&
is.na(confirm$pyramidalfss)==FALSE) {
              if(confirm$edss>=followup$edss &
confirm$pyramidalfss>=followup$pyramidalfss) {
                relapse_activity <-
subset(patient_relapses,patient_relapses$date>=confirm$date-30 &
patient_relapses$date<=confirm$date)
                if(nrow(relapse_activity)==0)
                  confirmed <- TRUE
              }
              break
            }
          }
        }
      }
      if(followup$edss>=6.0) {
        if(followup$edss-baseline$edss>=0.5) {
          # We may have a convert, but need to confirm with a confirmation
EDSS >=3 months
          confirmed <- FALSE
          for(k in (j+1):nrow(patient_visits)) {
            confirm <- patient_visits[k,]
            if(confirm$date-followup$date>=90 &&
is.na(confirm$pyramidalfss)==FALSE) {
              if(confirm$edss>=followup$edss &
confirm$pyramidalfss>=followup$pyramidalfss) {
                relapse_activity <-
subset(patient_relapses,patient_relapses$date>=confirm$date-30 &
patient_relapses$date<=confirm$date)
                if(nrow(relapse_activity)==0)
                  confirmed <- TRUE
                #else
                # print("Relapse")
              }
              break
            }
          }
        }
      }

      if(confirmed==TRUE) {
        if(msbasealgorithm_mstype=="RR" || msbasealgorithm_mstype=="RR2") {
          msbasealgorithm_spdate <- followup$date
          msbasealgorithm_edss <- followup$edss
        }
        msbasealgorithm_mstype <- "SP"
      }

    }
  }
```

```
  if(msbasealgorithm_mstype=="RR2") # If we still have RR2, this is
unclassifiable
    msbasealgorithm_mstype <- ""
  patients[patients$patientcode==patient,]$msbasealgorithmfs_mstype <<-
msbasealgorithm_mstype
  patients[patients$patientcode==patient,]$msbasealgorithmfs_spdate <<-
msbasealgorithm_spdate
  patients[patients$patientcode==patient,]$msbasealgorithmfs_edss <<-
msbasealgorithm_edss
}

# Get msbasealgorithm with FS

patients$msbasealgorithmfs_mstype <- ""
patients$msbasealgorithmfs_spdate <- as.Date(NA)
patients$msbasealgorithmfs_edss <- as.numeric(NA) #CORRECTED TO PREVENT
LATER ERROR WHEN ASSIGNING EXACT VALUE
print(sprintf("Getting MSBase classification with FS score"))
print(sprintf("Number of patients: %d",nrow(patients)))
for(row in 1:nrow(patients)) {
  if(row %% 100 == 0)
    print(sprintf("Patients: %d",row))
  patient <- patients[row,]
  msbaseAlgorithmFS(patient$patientcode,FALSE)
}

# Get Decision Tree
# DT1 = Decision Tree by scanning longitudinally (only way to get
conversion date)
# DT2 = Decision Tree by classifying index date (no way to get conversion
date)

patients$dt1_mstype <- ""
patients$dt2_mstype <- ""
patients$dt1_spdate <- as.Date(NA)
patients$dt1_edss <- as.numeric(NA)
print(sprintf("Getting Decision Tree classification"))
for(row in 1:nrow(patients)) {
  if(row %% 100 == 0)
    print(sprintf("Patients: %d",row))
  patient <- patients[row,]

  patient <- patients[row,]
  patient_visits <- subset(visits,visits$patientcode ==
patient$patientcode)
  patient_visits <- patient_visits[order(patient_visits$date),]

  dt1_mstype <- ""
  dt1_spdate <- as.Date(NA)
  dt1_edss <- NA

  for(i in 1:(nrow(patient_visits))) {
```

```
    age <- floor(as.numeric(patient_visits[i,]$date-patient$dateofbirth)/
365.25)
    edss <- patient_visits[i,]$edss

    # This is the longitudinal part of the analysis, i.e.
    # Decision Tree #1.
    if(dt1_mstype=="SP")
      next

    if(edss<2.8)
      dt1_mstype <- "RR"
    if(edss>=2.8 && edss<4.3 && age<56)
      dt1_mstype <- "RR"
    if(edss>=4.3 && edss<6.3 && age<45)
      dt1_mstype <- "RR"
    if(edss>=2.8 && edss<3.8 && age>=56 && age<64)
      dt1_mstype <- "RR"
    if(edss>=2.8 && edss<3.3 && age>=64)
      dt1_mstype <- "RR"
    if(edss>=3.8 && edss<4.3 && age>=56 && age<64) {
      if(dt1_mstype == "RR") {
        dt1_spdate <- patient_visits[i,]$date
        dt1_edss <- patient_visits[i,]$edss
      }
      dt1_mstype <- "SP"
    }
    if(edss>=3.3 && edss<4.3 && age>=64) {
      if(dt1_mstype == "RR") {
        dt1_spdate <- patient_visits[i,]$date
        dt1_edss <- patient_visits[i,]$edss
      }
      dt1_mstype <- "SP"
    }
    if(edss>=4.3 && edss<6.3 && age>=45) {
      if(dt1_mstype == "RR") {
        dt1_spdate <- patient_visits[i,]$date
        dt1_edss <- patient_visits[i,]$edss
      }
      dt1_mstype <- "SP"
    }
    if(edss>=6.3) {
      if(dt1_mstype == "RR") {
        dt1_spdate <- patient_visits[i,]$date
        dt1_edss <- patient_visits[i,]$edss
      }
      dt1_mstype <- "SP"
    }
  }

  # This is the cross-sectional part of the analysis, i.e.
  # Decision Tree #2.

  dt2_mstype <- ""
```

```
  if(edss<2.8)
    dt2_mstype <- "RR"
  if(edss>=2.8 && edss<4.3 && age<56)
    dt2_mstype <- "RR"
  if(edss>=4.3 && edss<6.3 && age<45)
    dt2_mstype <- "RR"
  if(edss>=2.8 && edss<3.8 && age>=56 && age<64)
    dt2_mstype <- "RR"
  if(edss>=2.8 && edss<3.3 && age>=64)
    dt2_mstype <- "RR"
  if(edss>=3.8 && edss<4.3 && age>=56 && age<64)
    dt2_mstype <- "SP"
  if(edss>=3.3 && edss<4.3 && age>=64)
    dt2_mstype <- "SP"
  if(edss>=4.3 && edss<6.3 && age>=45)
    dt2_mstype <- "SP"
  if(edss>=6.3)
    dt2_mstype <- "SP"

  patients[row,]$dt1_mstype <- dt1_mstype
  patients[row,]$dt1_spdate <- dt1_spdate
  patients[row,]$dt1_edss <- dt1_edss
  patients[row,]$dt2_mstype <- dt2_mstype
}

# Only include patients with SP-date for all methods
# Get categories of patients
patients$spdate <- patients$clinicalspdate
patients$edssconv <- patients$clinical_edss
clinicalSPMS <- subset(patients,patients$clinicalmstype=="SP")
clinicalRRMS <- subset(patients,patients$clinicalmstype=="RR")
clinicalNONE <- subset(patients,patients$clinicalmstype=="")

patients$mstype <- patients$expand_mstype
patients$spdate <- as.Date(patients$expand_spdate)
patients$edssconv <- patients$expand_edss
expandSPMS <- subset(patients,patients$expand_mstype=="SP")
expandRRMS <- subset(patients,patients$expand_mstype=="RR")
expandNONE <- subset(patients,patients$expand_mstype=="")

patients$mstype <- patients$msbasealgorithm_mstype
patients$spdate <- as.Date(patients$msbasealgorithm_spdate)
patients$edssconv <- patients$msbasealgorithm_edss
msbasealgorithmSPMS <-
subset(patients,patients$msbasealgorithm_mstype=="SP")
msbasealgorithmRRMS <-
subset(patients,patients$msbasealgorithm_mstype=="RR")
msbasealgorithmNONE <- subset(patients,patients$msbasealgorithm_mstype=="")

patients$mstype <- patients$msbasealgorithmfs_mstype
patients$spdate <- as.Date(patients$msbasealgorithmfs_spdate)
patients$edssconv <- patients$msbasealgorithmfs_edss
```

```
msbasealgorithmFS_SPMS <-
subset(patients,patients$msbasealgorithmfs_mstype=="SP")
msbasealgorithmFS_RRMS <-
subset(patients,patients$msbasealgorithmfs_mstype=="RR")
msbasealgorithmFS_NONE <-
subset(patients,patients$msbasealgorithmfs_mstype=="")


patients$mstype <- patients$dt1_mstype
patients$spdate <- as.Date(patients$dt1_spdate)
patients$edssconv <- patients$dt1_edss
dt1SPMS <- subset(patients,patients$dt1_mstype=="SP")
dt1RRMS <- subset(patients,patients$dt1_mstype=="RR")
dt1NONE <- subset(patients,patients$dt1_mstype=="")

patients$mstype <- patients$dt2_mstype
patients$spdate <- NA
patients$edssconv <- NA
dt2SPMS <- subset(patients,patients$dt2_mstype=="SP")
dt2RRMS <- subset(patients,patients$dt2_mstype=="RR")
dt2NONE <- subset(patients,patients$dt2_mstype=="")

patients$mstype <- patients$clinicalmstype

dmtsatindex <- sort(unique(patients$dmtnameatindex))
dmtsatindexcol1 <- c(dmtnames,"")
dmtnames <- c(dmtnames,"Missing")




# Create the table
column1 <- c("N","Gender","Male","Female","Missing","")
column1 <- c(column1,"Age at index date
(year)","Mean","SD","Median","Q1","Q3","Min","Max","")
column1 <- c(column1,"Age at conversion to SPMS
(year)","N","Mean","SD","Median","Q1","Q3","Min","Max","Missing","")
column1 <- c(column1,"Age at MS onset
(year)","N","Mean","SD","Median","Q1","Q3","Min","Max","Missing","")
column1 <- c(column1,"Time from MS onset to SPMS
(year)","N","Mean","SD","Median","Q1","Q3","Min","Max","Missing","")
column1 <- c(column1,"Time from MS diagnosis to SPMS
(year)","N","Mean","SD","Median","Q1","Q3","Min","Max","Missing","")
column1 <- c(column1,"Time from SPMS to index date
(year)","N","Mean","SD","Median","Q1","Q3","Min","Max","Missing","")
column1 <- c(column1,"EDSS score at SPMS conversion date (±12
months)","N","Mean","SD","Median","Q1","Q3","Min","Max","Missing","")
column1 <- c(column1,"EDSS score at index date (±12
months)","N","Mean","SD","Median","Q1","Q3","Min","Max","Missing","")
```

```
column1 <- c(column1,"N","Clinical SPMS","Clinical RRMS","Clinical SPMS
Proportion","Clinical RRMS proportion","EXPAND SPMS","EXPAND RRMS","EXPAND
Unclassifiable","MSBase algorithm SPMS","MSBase algorithm RRMS","MSBase
algorithm Unclassifiable")
column1 <- c(column1,"DT1 SPMS","DT1 RRMS","DT1 Unclassifiable","DT2
SPMS","DT2 RRMS","DT2 Unclassifiable","MSBase algorithm FS SPMS","MSBase
algorithm FS RRMS","MSBase algorithm FS Unclassifiable")
column1 <- c(column1,"N SPonset difference","Mean SPonset difference","SD
SPonset difference","")
column1 <- c(column1,"5 years EDSS count from
index","Mean","SD","Median","Q1","Q3","Min","Max","")
column1 <- c(column1,"5 years EDSS count from
conversion","N","Mean","SD","Median","Q1","Q3","Min","Max","Missing")

column1 <- c(column1,"","DMT at index")
column1 <- c(column1,dmtnames)
column1 <- c(column1,"","DMT at conversion","")
column1 <- c(column1,"Number of relapses during last 2-years prior to index
date","0","1","2","3","4+","")

column1 <- c(column1,"RRMS patients classified as SPMS:")
column1 <- c(column1,"N","Gender","Male","Female","Missing","")
column1 <- c(column1,"Age at index date
(year)","Mean","SD","Median","Q1","Q3","Min","Max","")
column1 <- c(column1,"Age at conversion to SPMS
(year)","N","Mean","SD","Median","Q1","Q3","Min","Max","Missing","")
column1 <- c(column1,"Age at MS onset
(year)","N","Mean","SD","Median","Q1","Q3","Min","Max","Missing","")
column1 <- c(column1,"Time from MS onset to SPMS
(year)","N","Mean","SD","Median","Q1","Q3","Min","Max","Missing","")
column1 <- c(column1,"Time from MS diagnosis to SPMS
(year)","N","Mean","SD","Median","Q1","Q3","Min","Max","Missing","")
column1 <- c(column1,"Time from SPMS to index date
(year)","N","Mean","SD","Median","Q1","Q3","Min","Max","Missing","")
column1 <- c(column1,"EDSS score at SPMS conversion date (±12
months)","N","Mean","SD","Median","Q1","Q3","Min","Max","Missing","")
column1 <- c(column1,"EDSS score at index
date","N","Mean","SD","Median","Q1","Q3","Min","Max","Missing","")
column1 <- c(column1,"N","Clinical SPMS","Clinical RRMS","Clinical SPMS
Proportion","Clinical RRMS proportion","EXPAND SPMS","EXPAND RRMS","EXPAND
Unclassifiable","MSBase algorithm SPMS","MSBase algorithm RRMS","MSBase
algorithm Unclassifiable")
column1 <- c(column1,"DT1 SPMS","DT1 RRMS","DT1 Unclassifiable","DT2
SPMS","DT2 RRMS","DT2 Unclassifiable","MSBase algorithm FS SPMS","MSBase
algorithm FS RRMS","MSBase algorithm FS Unclassifiable")
column1 <- c(column1,"N SPonset difference","Mean SPonset difference","SD
SPonset difference","")
column1 <- c(column1,"5 years EDSS count from
index","Mean","SD","Median","Q1","Q3","Min","Max","")
column1 <- c(column1,"5 years EDSS count from
conversion","N","Mean","SD","Median","Q1","Q3","Min","Max","Missing")
```

```
column1 <- c(column1,"","DMT at index")
column1 <- c(column1,dmtnames)
column1 <- c(column1,"","DMT at conversion","")
column1 <- c(column1,"Number of relapses during last 2-years prior to index
date","0","1","2","3","4+","")

column1 <- c(column1,"Version")

getResults <- function(msgroup) {
  results <- c()
  results[1]  <- nrow(msgroup)
  results[2]  <- ""
  results[3]  <- nrow(subset(msgroup,msgroup$gender=="M"))
  results[4]  <- nrow(subset(msgroup,msgroup$gender=="F"))
  results[5]  <- nrow(subset(msgroup,msgroup$gender==""))
  results[6]  <- ""

  # Age at index date
  results[7]  <- ""
  if(nrow(msgroup)>0) {
    results[8]  <- mean(msgroup$ageatindex)
    results[9]  <- sd(msgroup$ageatindex)
    results[10] <- median(msgroup$ageatindex)
    results[11] <- quantile(msgroup$ageatindex)[2]
    results[12] <- quantile(msgroup$ageatindex)[4]
    results[13] <- min(msgroup$ageatindex)
    results[14] <- max(msgroup$ageatindex)
  } else {
    results[8]  <- ""
    results[9]  <- ""
    results[10] <- ""
    results[11] <- ""
    results[12] <- ""
    results[13] <- ""
    results[14] <- ""
  }
  results[15] <- ""

  # Age at SPMS conversion
  results[16] <- ""
  tmpgroup <- subset(msgroup,is.na(msgroup$spdate)==FALSE)
  if(nrow(tmpgroup)>0) {
    ageatconversion <- as.numeric(tmpgroup$spdate-tmpgroup$dateofbirth)/
365.25
    results[17] <- nrow(subset(msgroup,is.na(msgroup$spdate)==FALSE))
    results[18] <- mean(ageatconversion)
    results[19] <- sd(ageatconversion)
    results[20] <- median(ageatconversion)
    results[21] <- quantile(ageatconversion)[2]
    results[22] <- quantile(ageatconversion)[4]
    results[23] <- min(ageatconversion)
    results[24] <- max(ageatconversion)
    results[25] <- nrow(subset(msgroup,is.na(msgroup$spdate)))
```

```
  } else {
    for(row in 17:25)
      results[row] <- ""
  }
  results[26] <- ""


  # Age at MS symptoms onset
  results[27] <- ""
  tmpgroup <- subset(msgroup,is.na(msgroup$onsetdate)==FALSE)
  if(nrow(tmpgroup)>0) {
    ageatonset <- as.numeric(tmpgroup$onsetdate-tmpgroup$dateofbirth)/
365.25
    results[28] <- nrow(subset(msgroup,is.na(msgroup$onsetdate)==FALSE))
    results[29] <- mean(ageatonset)
    results[30] <- sd(ageatonset)
    results[31] <- median(ageatonset)
    results[32] <- quantile(ageatonset)[2]
    results[33] <- quantile(ageatonset)[4]
    results[34] <- min(ageatonset)
    results[35] <- max(ageatonset)
    results[36] <- nrow(subset(msgroup,is.na(msgroup$onsetdate)))
  } else {
    for(row in 28:36)
      results[row] <- ""
  }
  results[37] <- ""

  # Time since MS onset to SPMS conversion
  results[38] <- ""
  tmpgroup <- subset(msgroup,is.na(msgroup$onsetdate)==FALSE &
is.na(msgroup$spdate)==FALSE)
  if(nrow(tmpgroup)>0) {
    yearsfromonset <- as.numeric(tmpgroup$spdate-tmpgroup$onsetdate)/365.25
    results[39] <- nrow(tmpgroup)
    results[40] <- mean(yearsfromonset)
    results[41] <- sd(yearsfromonset)
    results[42] <- median(yearsfromonset)
    results[43] <- quantile(yearsfromonset)[2]
    results[44] <- quantile(yearsfromonset)[4]
    results[45] <- min(yearsfromonset)
    results[46] <- max(yearsfromonset)
    results[47] <- nrow(msgroup)-nrow(tmpgroup)
  } else {
    for(row in 39:47)
      results[row] <- ""
  }
  results[48] <- ""

  # Time since MS diagnosis to SPMS conversion
  results[49] <- ""
  tmpgroup <- subset(msgroup,is.na(msgroup$diagnosisdate)==FALSE &
is.na(msgroup$spdate)==FALSE)
```

```
  if(nrow(tmpgroup)>0) {
    yearsfromdiagnosis <- as.numeric(tmpgroup$spdate-
tmpgroup$diagnosisdate)/365.25
    results[50] <- nrow(tmpgroup)
    results[51] <- mean(yearsfromdiagnosis)
    results[52] <- sd(yearsfromdiagnosis)
    results[53] <- median(yearsfromdiagnosis)
    results[54] <- quantile(yearsfromdiagnosis)[2]
    results[55] <- quantile(yearsfromdiagnosis)[4]
    results[56] <- min(yearsfromdiagnosis)
    results[57] <- max(yearsfromdiagnosis)
    results[58] <- nrow(msgroup)-nrow(tmpgroup)
  } else {
    for(row in 50:58)
      results[row] <- ""
  }
  results[59] <- ""

  # Time from SPMS conversion to index date
  results[60] <- ""
  tmpgroup <- subset(msgroup,is.na(msgroup$spdate)==FALSE)
  if(nrow(tmpgroup)>0) {
    yearsfromconversion <- as.numeric(tmpgroup$indexdate-tmpgroup$spdate)/
365.25
    results[61] <- nrow(tmpgroup)
    results[62] <- mean(yearsfromconversion)
    results[63] <- sd(yearsfromconversion)
    results[64] <- median(yearsfromconversion)
    results[65] <- quantile(yearsfromconversion)[2]
    results[66] <- quantile(yearsfromconversion)[4]
    results[67] <- min(yearsfromconversion)
    results[68] <- max(yearsfromconversion)
    results[69] <- nrow(msgroup)-nrow(tmpgroup)
  } else {
    for(row in 61:69)
      results[row] <- ""
  }
  results[70] <- ""

  # EDSS score at SPMS conversion date
  results[71] <- ""
  tmpgroup <- subset(msgroup,is.na(msgroup$edssconv)==FALSE)
  if(nrow(tmpgroup)>0) {
    edssconv <- tmpgroup$edssconv
    results[72] <- nrow(tmpgroup)
    results[73] <- mean(edssconv)
    results[74] <- sd(edssconv)
    results[75] <- median(edssconv)
    results[76] <- quantile(edssconv)[2]
    results[77] <- quantile(edssconv)[4]
    results[78] <- min(edssconv)
    results[79] <- max(edssconv)
    results[80] <- nrow(msgroup)-nrow(tmpgroup)
```

```
  } else {
    for(row in 72:80)
      results[row] <- ""
  }
  results[81] <- ""

  # EDSS score at index date
  results[82] <- ""
  tmpgroup <- msgroup
  if(nrow(tmpgroup)>0) {
    edss <- tmpgroup$indexedss
    results[83] <- nrow(tmpgroup)
    results[84] <- mean(edss)
    results[85] <- sd(edss)
    results[86] <- median(edss)
    results[87] <- quantile(edss)[2]
    results[88] <- quantile(edss)[4]
    results[89] <- min(edss)
    results[90] <- max(edss)
    results[91] <- nrow(msgroup)-nrow(tmpgroup)
  } else {
    for(row in 83:91)
      results[row] <- ""
  }

  results[92] <- ""
  results[93] <- nrow(msgroup)
  results[94] <- sum(msgroup$clinicalmstype=="SP")
  results[95] <- sum(msgroup$clinicalmstype=="RR")
  results[96] <- as.numeric(results[94])/as.numeric(results[93])
  results[97] <- as.numeric(results[95])/as.numeric(results[93])
  results[98] <- sum(msgroup$expand_mstype=="SP")
  results[99] <- sum(msgroup$expand_mstype=="RR")
  results[100] <- sum(msgroup$expand_mstype=="")
  results[101] <- sum(msgroup$msbasealgorithm_mstype=="SP")
  results[102] <- sum(msgroup$msbasealgorithm_mstype=="RR")
  results[103] <- sum(msgroup$msbasealgorithm_mstype=="")
  results[104] <- sum(msgroup$dt1_mstype=="SP")
  results[105] <- sum(msgroup$dt1_mstype=="RR")
  results[106] <- sum(msgroup$dt1_mstype=="")
  results[107] <- sum(msgroup$dt2_mstype=="SP")
  results[108] <- sum(msgroup$dt2_mstype=="RR")
  results[109] <- sum(msgroup$dt2_mstype=="")
  results[110] <- sum(msgroup$msbasealgorithmfs_mstype=="SP")
  results[111] <- sum(msgroup$msbasealgorithmfs_mstype=="RR")
  results[112] <- sum(msgroup$msbasealgorithmfs_mstype=="")

  tmpdata <- subset(msgroup,is.na(msgroup$clinicalspdate)==FALSE &
is.na(msgroup$spdate)==FALSE)
  tmpdata$spdate <- as.Date(tmpdata$spdate)
  tmpdata$clinicalspdate <- as.Date(tmpdata$clinicalspdate)
  results[113] <- nrow(tmpdata)
```

```
  results[114] <- mean(as.numeric(tmpdata$spdate-tmpdata$clinicalspdate)/
365.25)
  results[115] <- sd(as.numeric(tmpdata$spdate-tmpdata$clinicalspdate)/
365.25)
  results[116] <- ""

  # Number of previous EDSS scores five years back from index
  results[117] <- ""
  if(nrow(msgroup)>0) {
    results[118] <- mean(msgroup$indexedsscounts)
    results[119] <- sd(msgroup$indexedsscounts)
    results[120] <- median(msgroup$indexedsscounts)
    results[121] <- quantile(msgroup$indexedsscounts)[2]
    results[122] <- quantile(msgroup$indexedsscounts)[4]
    results[123] <- min(msgroup$indexedsscounts)
    results[124] <- max(msgroup$indexedsscounts)
  } else {
    for(row in 118:124)
      results[row] <- ""
  }

  results[125] <- ""

    # Number of previous EDSS scores five years back from conversion
  tmpgroup <- subset(msgroup,is.na(msgroup$spdate)==FALSE)
  results[126] <- ""
  if(nrow(tmpgroup)>0) {
    tmpgroup$conversionedsscounts <- 0
    for(row in 1:nrow(tmpgroup)) {
      spdate <- tmpgroup[row,]$spdate
      patient_visits <- subset(visits,visits$patientcode == tmpgroup[row,]
$patientcode)
      tmpgroup[row,]$conversionedsscounts <-
sum(patient_visits$date>=(spdate-365.25*5) & patient_visits$date<=spdate)
    }
    results[127] <- nrow(tmpgroup)
    results[128] <- mean(tmpgroup$conversionedsscounts)
    results[129] <- sd(tmpgroup$conversionedsscounts)
    results[130] <- median(tmpgroup$conversionedsscounts)
    results[131] <- quantile(tmpgroup$conversionedsscounts)[2]
    results[132] <- quantile(tmpgroup$conversionedsscounts)[4]
    results[133] <- min(tmpgroup$conversionedsscounts)
    results[134] <- max(tmpgroup$conversionedsscounts)
    results[135] <- nrow(msgroup)-nrow(tmpgroup)
  } else {
    for(row in 127:135)
      results[row] <- ""
  }
  results[136] <- ""
  results[137] <- sum(msgroup$dmtatindex)


  # DMT at index?
```

```
  numberofdmts <- length(dmtnames)
  for(row in 138:(138+numberofdmts-1))
    results[row] <- sum(msgroup$dmtnameatindex==dmtsatindexcol1[row-137])

  results[138+numberofdmts] <- ""

  if(nrow(msgroup)>0) {
    msgroup$dmtatconversion <- FALSE
    for(row in 1:nrow(msgroup)) {
      conversiondate <- msgroup[row,]$spdate
      if(is.na(conversiondate)==FALSE) {
        patient_dmts <- subset(dmt,dmt$patientcode == msgroup[row,]
$patientcode)
        patient_dmts <-
subset(patient_dmts,patient_dmts$startdate<=conversiondate &
(is.na(patient_dmts$stopdate)==TRUE |
patient_dmts$stopdate>=conversiondate))

        if(nrow(patient_dmts)>0)
          msgroup[row,]$dmtatconversion <- TRUE
      }
    }
  }

  results[138+numberofdmts+1] <- sum(msgroup$dmtatconversion)

  results[138+numberofdmts+2] <- "" # Empty line
  results[138+numberofdmts+3] <- "" # Number of relapses
  results[138+numberofdmts+4] <- sum(msgroup$indexrelapses==0) # 0
  results[138+numberofdmts+5] <- sum(msgroup$indexrelapses==1) # 1
  results[138+numberofdmts+6] <- sum(msgroup$indexrelapses==2) # 2
  results[138+numberofdmts+7] <- sum(msgroup$indexrelapses==3) # 3
  results[138+numberofdmts+8] <- sum(msgroup$indexrelapses>=4) # 4

  results[138+numberofdmts+9] <- "" # Empty line
  results[138+numberofdmts+10] <- "" #Section title

  # Now, take subgroup that become SP with the method but is RR clinically.
  msgroup <- subset(msgroup,msgroup$mstype=="SP" &
msgroup$clinicalmstype=="RR")
  startrow <- 138+numberofdmts+10

  results[1+startrow]  <- nrow(msgroup)
  results[2+startrow]  <- ""
  results[3+startrow]  <- nrow(subset(msgroup,msgroup$gender=="M"))
  results[4+startrow]  <- nrow(subset(msgroup,msgroup$gender=="F"))
  results[5+startrow]  <- nrow(subset(msgroup,msgroup$gender==""))
  results[6+startrow]  <- ""

  # Age at index date
  results[7+startrow]  <- ""
  if(nrow(msgroup)>0) {
    results[8+startrow]  <- mean(msgroup$ageatindex)
```

```
    results[9+startrow]  <- sd(msgroup$ageatindex)
    results[10+startrow] <- median(msgroup$ageatindex)
    results[11+startrow] <- quantile(msgroup$ageatindex)[2]
    results[12+startrow] <- quantile(msgroup$ageatindex)[4]
    results[13+startrow] <- min(msgroup$ageatindex)
    results[14+startrow] <- max(msgroup$ageatindex)
  } else {
    results[8+startrow]  <- ""
    results[9+startrow]  <- ""
    results[10+startrow]<- ""
    results[11+startrow]<- ""
    results[12+startrow]<- ""
    results[13+startrow]<- ""
    results[14+startrow]<- ""
  }
  results[15+startrow]<- ""

  # Age at SPMS conversion
  results[16+startrow]<- ""
  tmpgroup <- subset(msgroup,is.na(msgroup$spdate)==FALSE)
  if(nrow(tmpgroup)>0) {
    ageatconversion <- as.numeric(tmpgroup$spdate-tmpgroup$dateofbirth)/
365.25
    results[17+startrow]<-
nrow(subset(msgroup,is.na(msgroup$spdate)==FALSE))
    results[18+startrow]<- mean(ageatconversion)
    results[19+startrow]<- sd(ageatconversion)
    results[20+startrow]<- median(ageatconversion)
    results[21+startrow]<- quantile(ageatconversion)[2]
    results[22+startrow]<- quantile(ageatconversion)[4]
    results[23+startrow]<- min(ageatconversion)
    results[24+startrow]<- max(ageatconversion)
    results[25+startrow]<- nrow(subset(msgroup,is.na(msgroup$spdate)))
  } else {
    for(row in 17:25)
      results[row+startrow]<- ""
  }
  results[26+startrow]<- ""


  # Age at MS symptoms onset
  results[27+startrow]<- ""
  tmpgroup <- subset(msgroup,is.na(msgroup$onsetdate)==FALSE)
  if(nrow(tmpgroup)>0) {
    ageatonset <- as.numeric(tmpgroup$onsetdate-tmpgroup$dateofbirth)/
365.25
    results[28+startrow]<-
nrow(subset(msgroup,is.na(msgroup$onsetdate)==FALSE))
    results[29+startrow]<- mean(ageatonset)
    results[30+startrow]<- sd(ageatonset)
    results[31+startrow]<- median(ageatonset)
    results[32+startrow]<- quantile(ageatonset)[2]
    results[33+startrow]<- quantile(ageatonset)[4]
```

```
    results[34+startrow]<- min(ageatonset)
    results[35+startrow]<- max(ageatonset)
    results[36+startrow]<- nrow(subset(msgroup,is.na(msgroup$onsetdate)))
  } else {
    for(row in 28:36)
      results[row+startrow]<- ""
  }
  results[37+startrow]<- ""

  # Time since MS onset to SPMS conversion
  results[38+startrow]<- ""
  tmpgroup <- subset(msgroup,is.na(msgroup$onsetdate)==FALSE &
is.na(msgroup$spdate)==FALSE)
  if(nrow(tmpgroup)>0) {
    yearsfromonset <- as.numeric(tmpgroup$spdate-tmpgroup$onsetdate)/365.25
    results[39+startrow]<- nrow(tmpgroup)
    results[40+startrow]<- mean(yearsfromonset)
    results[41+startrow]<- sd(yearsfromonset)
    results[42+startrow]<- median(yearsfromonset)
    results[43+startrow]<- quantile(yearsfromonset)[2]
    results[44+startrow]<- quantile(yearsfromonset)[4]
    results[45+startrow]<- min(yearsfromonset)
    results[46+startrow]<- max(yearsfromonset)
    results[47+startrow]<- nrow(msgroup)-nrow(tmpgroup)
  } else {
    for(row in 39:47)
      results[row+startrow]<- ""
  }
  results[48+startrow]<- ""

  # Time since MS diagnosis to SPMS conversion
  results[49+startrow]<- ""
  tmpgroup <- subset(msgroup,is.na(msgroup$diagnosisdate)==FALSE &
is.na(msgroup$spdate)==FALSE)
  if(nrow(tmpgroup)>0) {
    yearsfromdiagnosis <- as.numeric(tmpgroup$spdate-
tmpgroup$diagnosisdate)/365.25
    results[50+startrow]<- nrow(tmpgroup)
    results[51+startrow]<- mean(yearsfromdiagnosis)
    results[52+startrow]<- sd(yearsfromdiagnosis)
    results[53+startrow]<- median(yearsfromdiagnosis)
    results[54+startrow]<- quantile(yearsfromdiagnosis)[2]
    results[55+startrow]<- quantile(yearsfromdiagnosis)[4]
    results[56+startrow]<- min(yearsfromdiagnosis)
    results[57+startrow]<- max(yearsfromdiagnosis)
    results[58+startrow]<- nrow(msgroup)-nrow(tmpgroup)
  } else {
    for(row in 50:58)
      results[row+startrow]<- ""
  }
  results[59+startrow]<- ""

  # Time from SPMS conversion to index date
```

```
  results[60+startrow]<- ""
  tmpgroup <- subset(msgroup,is.na(msgroup$spdate)==FALSE)
  if(nrow(tmpgroup)>0) {
    yearsfromconversion <- as.numeric(tmpgroup$indexdate-tmpgroup$spdate)/
365.25
    results[61+startrow]<- nrow(tmpgroup)
    results[62+startrow]<- mean(yearsfromconversion)
    results[63+startrow]<- sd(yearsfromconversion)
    results[64+startrow]<- median(yearsfromconversion)
    results[65+startrow]<- quantile(yearsfromconversion)[2]
    results[66+startrow]<- quantile(yearsfromconversion)[4]
    results[67+startrow]<- min(yearsfromconversion)
    results[68+startrow]<- max(yearsfromconversion)
    results[69+startrow]<- nrow(msgroup)-nrow(tmpgroup)
  } else {
    for(row in 61:69)
      results[row+startrow]<- ""
  }
  results[70+startrow]<- ""

  # EDSS score at SPMS conversion date
  results[71+startrow]<- ""
  tmpgroup <- subset(msgroup,is.na(msgroup$edssconv)==FALSE)
  if(nrow(tmpgroup)>0) {
    edssconv <- tmpgroup$edssconv
    results[72+startrow]<- nrow(tmpgroup)
    results[73+startrow]<- mean(edssconv)
    results[74+startrow]<- sd(edssconv)
    results[75+startrow]<- median(edssconv)
    results[76+startrow]<- quantile(edssconv)[2]
    results[77+startrow]<- quantile(edssconv)[4]
    results[78+startrow]<- min(edssconv)
    results[79+startrow]<- max(edssconv)
    results[80+startrow]<- nrow(msgroup)-nrow(tmpgroup)
  } else {
    for(row in 72:80)
      results[row+startrow]<- ""
  }
  results[81+startrow]<- ""

  # EDSS score at index date
  results[82+startrow]<- ""
  tmpgroup <- msgroup
  if(nrow(tmpgroup)>0) {
    edss <- tmpgroup$indexedss
    results[83+startrow]<- nrow(tmpgroup)
    results[84+startrow]<- mean(edss)
    results[85+startrow]<- sd(edss)
    results[86+startrow]<- median(edss)
    results[87+startrow]<- quantile(edss)[2]
    results[88+startrow]<- quantile(edss)[4]
    results[89+startrow]<- min(edss)
    results[90+startrow]<- max(edss)
```

```
    results[91+startrow]<- nrow(msgroup)-nrow(tmpgroup)
  } else {
    for(row in 83:91)
      results[row+startrow]<- ""
  }


  results[92+startrow]<- ""
  results[93+startrow]<- nrow(msgroup)
  results[94+startrow]<- sum(msgroup$clinicalmstype=="SP")
  results[95+startrow]<- sum(msgroup$clinicalmstype=="RR")
  results[96+startrow]<- as.numeric(results[94+startrow])/
as.numeric(results[93+startrow])
  results[97+startrow]<- as.numeric(results[95+startrow])/
as.numeric(results[93+startrow])
  results[98+startrow]<- sum(msgroup$expand_mstype=="SP")
  results[99+startrow]<- sum(msgroup$expand_mstype=="RR")
  results[100+startrow]<- sum(msgroup$expand_mstype=="")
  results[101+startrow]<- sum(msgroup$msbasealgorithm_mstype=="SP")
  results[102+startrow]<- sum(msgroup$msbasealgorithm_mstype=="RR")
  results[103+startrow]<- sum(msgroup$msbasealgorithm_mstype=="")
  results[104+startrow]<- sum(msgroup$dt1_mstype=="SP")
  results[105+startrow]<- sum(msgroup$dt1_mstype=="RR")
  results[106+startrow]<- sum(msgroup$dt1_mstype=="")
  results[107+startrow]<- sum(msgroup$dt2_mstype=="SP")
  results[108+startrow]<- sum(msgroup$dt2_mstype=="RR")
  results[109+startrow]<- sum(msgroup$dt2_mstype=="")
  results[110+startrow]<- sum(msgroup$msbasealgorithmfs_mstype=="SP")
  results[111+startrow]<- sum(msgroup$msbasealgorithmfs_mstype=="RR")
  results[112+startrow]<- sum(msgroup$msbasealgorithmfs_mstype=="")

  tmpdata <- subset(msgroup,is.na(msgroup$clinicalspdate)==FALSE &
is.na(msgroup$spdate)==FALSE)
  tmpdata$spdate <- as.Date(tmpdata$spdate)
  tmpdata$clinicalspdate <- as.Date(tmpdata$clinicalspdate)
  results[113+startrow] <- nrow(tmpdata)
  results[114+startrow] <- mean(as.numeric(tmpdata$spdate-
tmpdata$clinicalspdate)/365.25)
  results[115+startrow] <- sd(as.numeric(tmpdata$spdate-
tmpdata$clinicalspdate)/365.25)

  results[116+startrow] <- ""

  # Number of previous EDSS scores five years back from index
  results[117+startrow] <- ""
  if(nrow(msgroup)>0) {
    results[118+startrow] <- mean(msgroup$indexedsscounts)
    results[119+startrow] <- sd(msgroup$indexedsscounts)
    results[120+startrow] <- median(msgroup$indexedsscounts)
    results[121+startrow] <- quantile(msgroup$indexedsscounts)[2]
    results[122+startrow] <- quantile(msgroup$indexedsscounts)[4]
    results[123+startrow] <- min(msgroup$indexedsscounts)
    results[124+startrow] <- max(msgroup$indexedsscounts)
```

```
  } else {
    for(row in 118:124)
      results[row+startrow] <- ""
  }

  results[125+startrow] <- ""

  # Number of previous EDSS scores five years back from conversion
  tmpgroup <- subset(msgroup,is.na(msgroup$spdate)==FALSE)
  results[126+startrow] <- ""
  if(nrow(tmpgroup)>0) {
    tmpgroup$conversionedsscounts <- 0
    for(row in 1:nrow(tmpgroup)) {
      spdate <- tmpgroup[row,]$spdate
      patient_visits <- subset(visits,visits$patientcode == tmpgroup[row,]
$patientcode)
      tmpgroup[row,]$conversionedsscounts <-
sum(patient_visits$date>=(spdate-365.25*5) & patient_visits$date<=spdate)
    }
    results[127+startrow] <- nrow(tmpgroup)
    results[128+startrow] <- mean(tmpgroup$conversionedsscounts)
    results[129+startrow] <- sd(tmpgroup$conversionedsscounts)
    results[130+startrow] <- median(tmpgroup$conversionedsscounts)
    results[131+startrow] <- quantile(tmpgroup$conversionedsscounts)[2]
    results[132+startrow] <- quantile(tmpgroup$conversionedsscounts)[4]
    results[133+startrow] <- min(tmpgroup$conversionedsscounts)
    results[134+startrow] <- max(tmpgroup$conversionedsscounts)
    results[135+startrow] <- nrow(msgroup)-nrow(tmpgroup)
  } else {
    for(row in 127:135)
      results[row+startrow] <- ""
  }
  #results[133+startrow] <- ""
  results[136+startrow] <- ""
  results[137+startrow] <- sum(msgroup$dmtatindex)

  if(nrow(msgroup)>0) {
    msgroup$dmtatconversion <- FALSE
    for(row in 1:nrow(msgroup)) {
      conversiondate <- msgroup[row,]$spdate
      if(is.na(conversiondate)==FALSE) {
        patient_dmts <- subset(dmt,dmt$patientcode == msgroup[row,]
$patientcode)
        patient_dmts <-
subset(patient_dmts,patient_dmts$startdate<=conversiondate &
(is.na(patient_dmts$stopdate)==TRUE |
patient_dmts$stopdate>=conversiondate))

        if(nrow(patient_dmts)>0)
          msgroup[row,]$dmtatconversion <- TRUE
      }
    }
  }
```

```
  # DMT at index?
  numberofdmts <- length(dmtnames)
  for(row in 138:(138+numberofdmts-1))
    results[startrow+row] <-
sum(msgroup$dmtnameatindex==dmtsatindexcol1[row-137])

  results[startrow+138+numberofdmts] <- ""

  if(nrow(msgroup)>0) {
    msgroup$dmtatconversion <- FALSE
    for(row in 1:nrow(msgroup)) {
      conversiondate <- msgroup[row,]$spdate
      if(is.na(conversiondate)==FALSE) {
        patient_dmts <- subset(dmt,dmt$patientcode == msgroup[row,]
$patientcode)
        patient_dmts <-
subset(patient_dmts,patient_dmts$startdate<=conversiondate &
(is.na(patient_dmts$stopdate)==TRUE |
patient_dmts$stopdate>=conversiondate))

        if(nrow(patient_dmts)>0)
          msgroup[row,]$dmtatconversion <- TRUE
      }
    }
  }

  results[startrow+138+numberofdmts+1] <- sum(msgroup$dmtatconversion)

  results[startrow+138+numberofdmts+2] <- "" # Empty line
  results[startrow+138+numberofdmts+3] <- "" # Number of relapses
  results[startrow+138+numberofdmts+4] <- sum(msgroup$indexrelapses==0) # 0
  results[startrow+138+numberofdmts+5] <- sum(msgroup$indexrelapses==1) # 1
  results[startrow+138+numberofdmts+6] <- sum(msgroup$indexrelapses==2) # 2
  results[startrow+138+numberofdmts+7] <- sum(msgroup$indexrelapses==3) # 3
  results[startrow+138+numberofdmts+8] <- sum(msgroup$indexrelapses>=4) # 4

  results[startrow+138+numberofdmts+9] <- "" # Empty line


  results[startrow+138+numberofdmts+10]<- "" # Version number

  return(results)
}

msgroup <- expandSPMS
x <- getResults(expandSPMS)

table1 <- data.frame(matrix(ncol=19,nrow=length(column1)))
table1[,1] <- column1
table1[,2] <- getResults(clinicalSPMS)
table1[,3] <- getResults(clinicalRRMS)
table1[,4] <- getResults(clinicalNONE)
```

```
table1[,5] <- getResults(expandSPMS)
table1[,6] <- getResults(expandRRMS)
table1[,7] <- getResults(expandNONE)
table1[,8] <- getResults(msbasealgorithmSPMS)
table1[,9] <- getResults(msbasealgorithmRRMS)
table1[,10] <- getResults(msbasealgorithmNONE)
table1[,11] <- getResults(dt1SPMS)
table1[,12] <- getResults(dt1RRMS)
table1[,13] <- getResults(dt1NONE)
table1[,14] <- getResults(dt2SPMS)
table1[,15] <- getResults(dt2RRMS)
table1[,16] <- getResults(dt2NONE)
table1[,17] <- getResults(msbasealgorithmFS_SPMS)
table1[,18] <- getResults(msbasealgorithmFS_RRMS)
table1[,19] <- getResults(msbasealgorithmFS_NONE)
table1[nrow(table1),2] <- version

colnames(table1) <- c("Characteristics","Clinical SPMS","Clinical
RRMS","Clinical unclassifiable","EXPAND SPMS","EXPAND RRMS","EXPAND
unclassifiable","MSBase Algorithm SPMS","MSBase algorithm RRMS","MSBase
algorithm unclassifiable","DT1 SPMS","DT1 RRMS","DT1 unclassifiable","DT2
SPMS","DT2 RRMS","DT2 unclassifiable","MSBase Algorithm FS SPMS","MSBase
algorithm FS RRMS","MSBase algorithm FS unclassifiable")
```
